# Recent trends in insect gut immunity

**DOI:** 10.3389/fimmu.2023.1272143

**Published:** 2023-12-18

**Authors:** Shahidul Ahmed Khan, Maryam Ali Mohmmadie Kojour, Yeon Soo Han

**Affiliations:** ^1^ Department of Applied Biology, Institute of Environmentally Friendly Agriculture (IEFA), College of Agriculture and Life Sciences, Chonnam National University, Gwangju, Republic of Korea; ^2^ Life & Medical Sciences Institute (LIMES) Development, Genetics & Molecular Physiology Unit, University of Bonn, Bonn, Germany

**Keywords:** gut immunity, gut compartments, cellular composition, microbiota, immune signaling pathways, *Drosophila melanogaster*, *Tenebrio molitor*, antimicrobial peptides

## Abstract

The gut is a crucial organ in insect defense against various pathogens and harmful substances in their environment and diet. Distinct insect gut compartments possess unique functionalities contributing to their physiological processes, including immunity. The insect gut’s cellular composition is vital for cellular and humoral immunity. The peritrophic membrane, mucus layer, lumen, microvilli, and various gut cells provide essential support for activating and regulating immune defense mechanisms. These components also secrete molecules and enzymes that are imperative in physiological activities. Additionally, the gut microbiota initiates various signaling pathways and produces vitamins and minerals that help maintain gut homeostasis. Distinct immune signaling pathways are activated within the gut when insects ingest pathogens or hazardous materials. The pathway induced depends on the infection or pathogen type; include immune deficiency (imd), Toll, JAK/STAT, Duox-ROS, and JNK/FOXO regulatory pathways. These pathways produce different antimicrobial peptides (AMPs) and maintain gut homeostasis. Furthermore, various signaling mechanisms within gut cells regulate insect gut recovery following infection. Although some questions regarding insect gut immunity in different species require additional study, this review provides insights into the insect gut’s structure and composition, commensal microorganism roles in *Drosophila melanogaster* and *Tenebrio molitor* life cycles, different signaling pathways involved in gut immune systems, and the insect gut post-infection recovery through various signaling mechanisms.

## 1 Introduction

Food is vital for life. To thrive in their natural environment and ensure survival, insects must obtain nourishment through various ways such as adaptations, physiological processes, and reproductive activities. Consequently, insects are compelled to gather food from diverse environmental outlets that often harbor harmful microorganisms that can invade the insect’s digestive system. Therefore, insect digestive systems must employ expansive defense mechanisms to counteract infection risks or potential harm (1).

The insect gut composition is crucial for orchestrating defense mechanisms. The peritrophic membrane, comprising chitin and protein, collaborates with glycosylated protein-based mucus, intestinal epithelial cells, and intestinal stem cells to establish a robust physical shield against invading pathogenic microorganisms (2). The peritrophic membrane is the foremost defense and a formidable barrier that safeguards the midgut epithelium from abrasive food particles and lethal pathogen incursions (3). The mucosal layer, enriched with mucins, concurrently lubricates the luminal surface and fortifies the epithelium, impeding mechanical damage, pathogens, and toxic molecules (4). Given an insect’s diverse dietary repertoire of decaying plants, fungal matter, and pathogenic bacteria, these organisms have evolved an impressive digestive enzyme arsenal for efficient carbohydrate, protein, and lipid processing for complex food sources. Intricate intestinal pH regulation is also pivotal in the initial food digestion stages, with a pH of 5 in the foregut, 7 to 9 in the midgut, and 5 in the hindgut. However, these pH fluctuations physically and chemically affect ingested food material, influencing its overall integrity (1).

In the face of pathogen attacks on host gut epithelial cells are tackled with a pathogen outbreak, cascades of immune responses are triggered, involving the activation of key immune genes. Notably, pattern recognition receptors (PRRs) and antimicrobial peptides (AMPs) are among the immune factors that spring into action. The immune deficiency pathway, orchestrated by the Nuclear Factor-kappaB (NF-kB) transcription factors REL2 and REL2-F, is stimulated, bolstering the host’s defense mechanisms (5). Furthermore, the Dualoxidase (Duox) pathway works diligently as another line of defense to maintain gut stability by safeguarding gut mucosa against reactive oxygen species (ROS)-sensitive pathogens (6). Moreover, the intricate protein interplay, including peptidoglycan receptor proteins (PGRPs), serine proteases, serpins, and a range of other molecules, reinforces the immune bolstering process through melanization within the prophenoloxidase (PPO) pathway, which varies in insect species (7). Additionally, the Janus kinase (JAK/STAT) signaling pathway, responsible for regulating cell growth, differentiation, apoptosis, and inflammatory reactions, triggers diverse antimicrobial peptide production, thereby instigating potent intestinal immunity in insects (8).

Various microorganism types have established symbiotic relationships with their hosts throughout evolution, providing extensive support. These microorganisms, commonly known as “commensal/microbiota,” are integral for enhancing host fecundity, development, and growth. Specifically, they contribute to food digestion, vitamin production, host gut defense against pathogens, harmful substance detoxification, and stimulating host immune responses (9). Gut microbiota has garnered recognition as a pivotal component for biological control and waste biodegradation while deterring insect-borne disease transmission (9). Furthermore, the gut microbiota provides significant functionalities in synthesizing proteins, catecholamine cross-linkers, and chitin, inducing various immune regulatory pathways to aid recovery from infection (10). The gut microbiota supports host defense by modulating pH and digestive enzyme levels. They actively compete with pathogens for resources, such as living space and nutrients, while producing antimicrobial substances (11).

This review highlights contemporary advancements in understanding the insect gut’s immune mechanisms when combating pathogenic threats. Specifically, we delve into the physical, biochemical, and intricate gut immune responses observed in model organisms, such as *D. melanogaster* and *T. molitor*, and further insight into post-infection recovery processes.

## 2 Insect gut structure and function

### 2.1 Insect gut compartments

**Figure 1:**
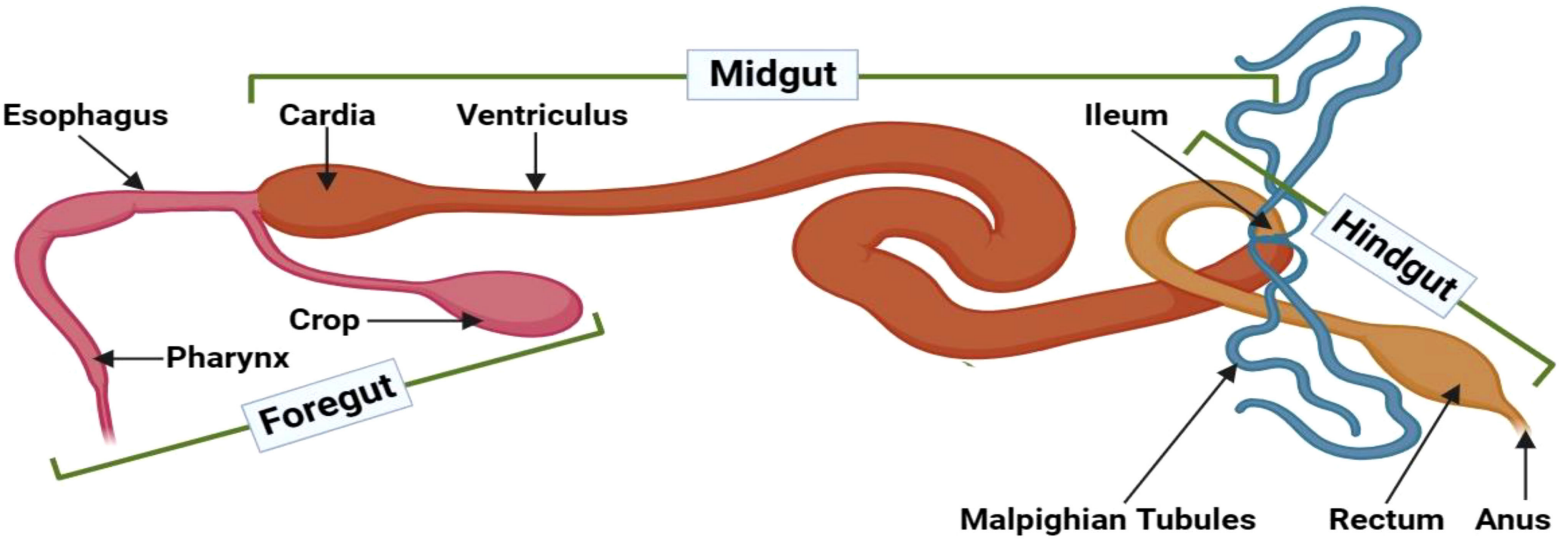
Gut compartments of adult *D. melanogaster*. The insect intestinal tract can be anatomically divided into three distinct compartments: the foregut, midgut, and hindgut. The foregut encompasses vital structures, such as the mouth, pharynx, esophagus, crop, and proventriculus, with robust cuticles that form a protective barrier against invading pathogens. Moreover, the foregut facilitates enzymatic compound secretions from the buccal cavity and salivary glands. The midgut begins at the gastric caeca and segments into anterior, median, and posterior regions, determined by their respective lengths. This section is notable for its relatively high permeability. The midgut’s epithelial lining incorporates diverse cell types with crucial functions, including nutrient digestion and absorption, pH regulation, endocrine modulation, epithelial growth, and safeguarding against pathogenic threats. Lastly, the hindgut starts where the Malpighian tubules and the muscular pyloric sphincter connect, followed by the ileum, colon, and rectum. Distinctive physiological processes in this compartment primarily focus on reabsorbing water, electrolytes, and other beneficial substances from the excreta. This illustration has been adopted from *T. H. Napoleao et al* (12).

The insect intestinal tract maintains a compartmentalized organization comprising three distinct sections: the foregut (stomodeum), midgut (mesenteron), and hindgut (proctodeum) (**Figure 1**). The foregut encompasses anatomical components, including the mouth, pharynx, esophagus, crop, and proventriculus. The midgut’s peritrophic matrix and mucins compose a formidable physical barrier, impeding pathogen intrusion. Lastly, the hindgut is essential for nutrient absorption and selective superfluous material exclusion (13).

#### 2.1.1 Foregut

The foregut represents the insect gut threshold, featuring an impermeable cuticle that imparts mechanical and chemical digestion properties facilitated by enzymes secreted from the buccal cavity. This section comprises salivary glands, reservoirs, and stomodeal valves that direct food flow toward the midgut (12). The crop is also integral to this region as it provides initial food storage, while the proventriculus acts as a valve that modifies ingested foodstuff (14). Certain insect species, such as *Carpenter bees*, symbiotically associate with *Lactobacillus* and *Enterobacteriaceae* bacteria predominantly located within the foregut. These microorganisms give support the bees’ digestive processes, nutrient delivery, pathogen defense, and immune signaling mechanisms (15). Moreover, the foregut in *Bulbitermes spp* is a site for xenobiotic degradation and initial metabolic processes (16). In *Anopheles culicifacies*, the foregut’s salivary gland harbors a more diverse symbiotic microorganism array than the midgut and hindgut, responsible for food acquisition, ingestion, and digestion (17). Research on *Cephalotes rohweri* indicates that the proventriculus valve, positioned between the crop and midgut, filters and effectively prevents pathogenic bacteria and particles larger than 0.2 µm from entering, protecting the host and maintaining host-microbe fidelity (18). Nutrient uptake and digestion are vital physiological processes across various life forms, and the gut-brain axis ensures gut homeostasis by regulating these processes through a neurohumoral communication system. Different neuropeptide classes govern the release of digestive enzymes, muscle activity, and engorgement, with specific neuropeptides presenting dynamic contributions to ecological pest management (19). Notably, *Drosophila* spp.*’s* gastric valve and crop function analogously to the stomach. A study investigating *Drosophila* stomach stem cells unveiled that JAK-STAT signaling regulates stem cell proliferation, Wingless signaling controls self-renewal, and hedgehog signaling governs cellular differentiation (20).

#### 2.1.2 Midgut

The midgut is a crucial component of the insect’s digestive tract that starts from the gastric caeca and serves as a primary site for digestion and nutrient absorption (12). Structurally, the midgut is divided into anterior, median, and posterior regions based on length (21). Comprising a diverse cell array, the midgut epithelium digests and absorbs nutrients and maintains luminal pH levels, endocrine regulation, and epithelial growth in this middle region (22). Within specific regions of this organ, messenger RNA expression governs enzyme secretion tailored for breaking down specific food components, such as proteins, carbohydrates, and lipids. These enzymes, along with the luminal pH, significantly contribute to eradicating pathogenic microorganisms that are ingested with food (22). Moreover, the midgut epithelium employs various secretion methods, including merocrine, apocrine, and micro-apocrine mechanisms. Diverse symbiotic microorganisms reside in the anterior midgut’s endoperitrophic space, assisting for digestion, and prompting immune responses (21). Regarding *Drosophila*, the midgut encompasses 14 distinct subregions that manifest distinct morphological, histological, and genetic properties. Damage to this compartment disrupts gut homeostasis, triggers stem cell proliferation, and stimulates various immune signaling pathways (23). When *Nasutitermes takasagoensis* digests cellulose, both endogenous endo-b-1,4-glucanase and b-glucosidase genes are uniformly expressed in the midgut, ensuring consistent enzyme production (24). In *Locusta migratoria*, the lethal giant larvae (Lgl) protein facilitates midgut morphology maintenance and ds-RNA-based insecticide development (25). Throughout *Drosophila* spp.*’s* lifespan, numerous regulatory signaling pathways operate within the midgut: the Delta/Notch signaling pathway regulates midgut progenitor cell differentiation, the Wingless and Int-1 (WNT) signaling pathway is responsible for intestinal stem cell (ISC) proliferation and maintenance, the Cytokine/Jak/Stat and Epidermal Growth Factor Receptor (EGFR) signaling pathways govern ISC proliferation, the JNK signaling pathway coordinates stress response and ISC proliferation; the Hippo/Salvador/Warts signaling pathway regulates midgut regeneration; and the cellular redox state influences ISC proliferation (26).

#### 2.1.3 Hindgut

The insect hindgut is structurally divided into the ileum, colon, and rectum, beginning at the pyloric valve after the midgut. Malpighian tubules in this region establish the excretory organs responsible for removing nitrogenous waste as uric acid from the hemolymph. This gut section is entirely covered by epithelial cells, with a muscular layer surrounding the basal membrane. The hindgut sustains greater permeability than the foregut, primarily absorbs nutrients and minerals, and eliminates feces (12). Additionally, the hindgut facilitates salt and amino acid reabsorption to maintain the hemolymph’s osmotic balance (27). Prophenoloxidase is a notable melanin production enzyme and serves as an immune response element at wound sites, functioning as the last line of defense against pathogens (26). The hindgut also harbors enteric bacteria, such as *Pantoea agglomerans*, which induce diverse immune responses (14).

### 2.2 The insect gut’s general cellular composition

**Figure 2:**
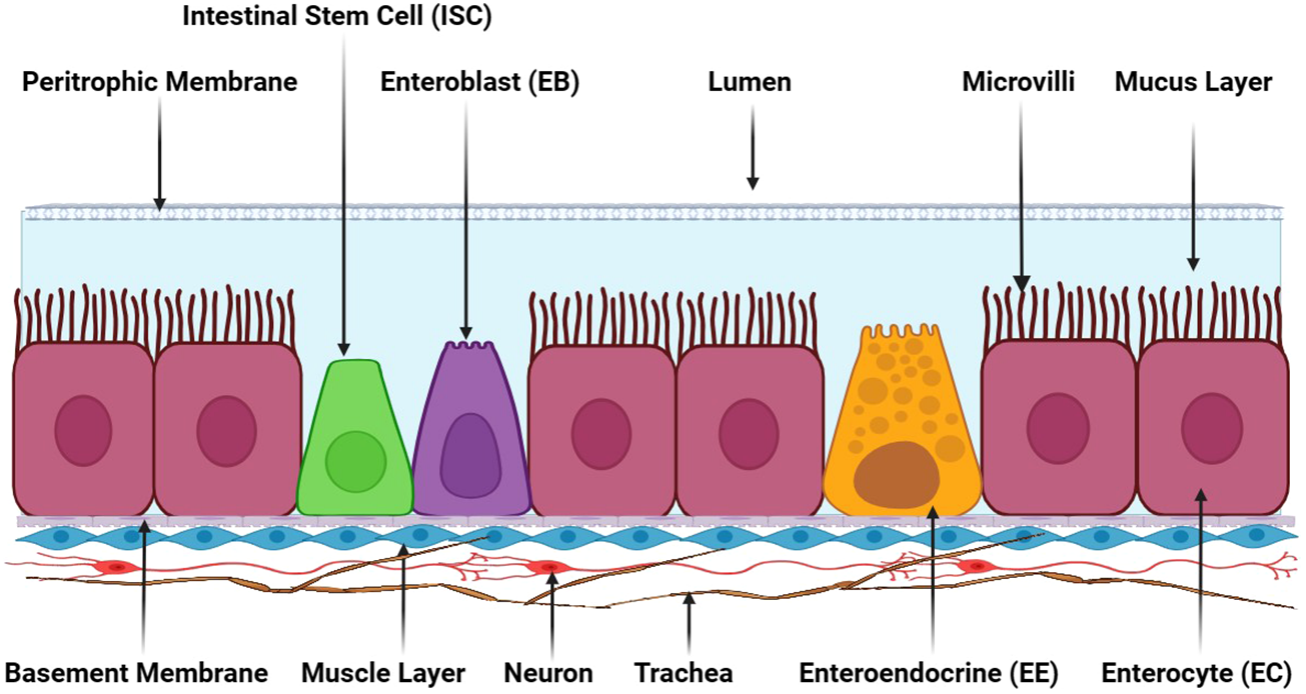
The adult insect gut’s general cellular composition. Adult insect gastrointestinal tracts comprise various cell types, namely absorptive enterocytes (ECs), secretory enteroendocrine cells (EEs), pluripotent intestinal stem cells (ISCs), and enteroblasts (EBs). These cells are collectively essential for an insect’s digestive process. The peritrophic membrane situated between the lumen and epithelial cells is a semipermeable non-cellular structure that serves as a protective barrier, shielding enterocytes from abrasive particles and invading pathogens. Adjacent to the peritrophic membrane lies a mucus layer that further engenders a protective environment for enterocyte cells. Beneath epithelial cells’ basement membranes is a muscle cell layer responsible for mechanical movements and contractions in the gut. Positioned underneath the muscle layer, neurons and trachea are present, providing innervation and oxygen supply to the gastrointestinal tract. This illustration has been adopted from *T. Kuraishi et al* (28).

The insect intestinal system comprises a myriad of cell types, including absorptive enterocytes, secretory enteroendocrine cells, and pluripotent intestinal stem cells. Additionally, muscle cells are situated beneath epithelial cells’ basement membranes (**Figure 2**). A semipermeable non-cellular structure, known as the peritrophic matrix/membrane, is positioned between the lumen and the epithelial layer within the insect gut, protecting enterocytes against abrasive particles and pathogens. Furthermore, a mucus layer between the peritrophic matrix and the enterocytes extends throughout the gut (28). Neurons and trachea are intricately integrated into the muscle layer beneath intestinal cells’ basement membrane, aiding the insect intestinal system’s overall functionality and coordination (1).

#### 2.2.1 Peritrophic membrane

The peritrophic membrane (PM) serves as a protective lining within the insect gut that separates the digestive compartment from the midgut lumen. Its primary functions include luminal surface lubrication to safeguard against food abrasion and pathogen invasion, as well as regulating immune responses. This membrane is composed of chitin fibers cross-linked by chitin-binding PM proteins (PMP); the PM forms a three-dimensional meshwork with water-filled pores. The mucin-like PMPs contain extensively O-glycosylated linker and chitin-binding domains (ChtBD2). In particular, these O-glycans are crucial for lubrication and controlling exclusion (29). Chitin and glycoprotein*s* form the intestinal PM, which activates various regulatory pathways to defend against pathogens and prevent gut tissue damage caused by pore-forming toxins (3). For example, the peritrophic membrane in mosquitos is a vital gut homeostasis regulator and immune response mediator (30). Furthermore, Heme-dependent peroxidase 1 (HPx1) promotes PM assembly and enhances antioxidant capacity while modulating vector competence. The *E75* transcription factor induces transcriptional *HPx1* activation, and *HPx1* knockdown accelerates DUOX-NADPH oxidase’s gut reactive oxygen species (ROS) production, thereby enhancing the immunological sensing of microbial infection (31). The peritrophic membrane of insects, exposed mucins and non-mucin protein receptors, functions as a binding site for various insect pathogens, such as *Junonia coenia densovirus* (*JcDV*) (32). Different protein classes within the insect PM exhibit distinct activities: Class I PM proteins contribute to PM integrity and function; Class II proteins faintly interact with the PM through adsorption; Class III proteins are released under harsher conditions; and Class IV proteins are covalently linked to other PM proteins or chitin, making their removal challenging (33). Seventeen peritrophic membrane proteins have been identified in the *Manduca sexta* midgut, and genes encoding peritrophic matrix proteins in *Tribolium castaneum* are directly associated with insect survival (34).

#### 2.2.2 Mucus layer and mucin

Mucus production is mediated by mucus-forming mucins (Mf-mucins). These proteins are glycosylated and form a protective layer on epithelial cells that line the respiratory, digestive, and urogenital tracts in vertebrates. Their primary function is to protect the organ cell lining against infections, dehydration, physical or chemical damage, and other threats while facilitating nutrient flow through the intestinal tract. In particular, studies have evidenced that the mucus layer is involved in Mf-mucins activation (35). While the mucus layer’s composition varies by organism, certain molecules remain consistent, particularly proteins extensively modified with N-acetylgalactosamine-based sugars attached to serines or threonines through O-linked glycosylation. Mucin proteins and their constituent O-glycans are fundamental for protecting and maintaining internal epithelial tissue vitality (36). In female insects, mucin proteins are localized in swollen crypts and are associated with symbiotic bacteria that stimulate gut immune responses (37).

#### 2.2.3 Microvilli

Microvilli form the inner intestinal cell surface and are intricately linked to the mucus layer. Microvilli contains jelly-associated enzymes composed of membrane-bound material. These enzymes are either bound to the cell membrane or entrapped within the glycocalyx and predicate microvilli formation and structure. The glycocalyx refers to the carbohydrate chain connected to integral proteins and glycolipids on the microvilli. Digestion primarily transpires on the microvilli surface, and insights into microvillar enzymology are invaluable for proteomics studies (38). Moreover, digestive enzymes are synthesized in the rough endoplasmic reticulum, processed in the Golgi complex, and packaged into secretory vesicles. Gut microvillar membranes’ physiological operations vary among insect taxa, influencing digestion, absorption, ion homeostasis, signaling, and secretory mechanisms. For instance, hemipteran gut cells manage various secretion types, including exocytic, apocrine, micro-apocrine with budding or pinched-off vesicles, and modified exocytic secretions (39).

#### 2.2.4 Insect gut cells

Enterocytes (ECs), enteroendocrine cells (EEs), ISCs, and enteroblasts (EBs) are the key cellular components involved in regulating the various activities and immune responses within the insect gut. Enterocytes primarily participate in absorption, whereas enteroendocrine cells secrete a diverse array of secretory molecules. ISCs are crucial for intestinal epithelium regeneration influenced by intrinsic factors in addition local and systemic stimuli (40). ISCs continuously generate progenitor cells that differentiate into multiple cell types within the epithelium, thereby maintaining homeostasis (41). Enteroblasts are transient progenitors that differentiate into ECs (42).

## 3 Insect gut microbiota

### 3.1 Gut microbiota in *D. melanogaster*

An insect encounters diverse environmental conditions and various foodstuffs harboring assorted microorganisms throughout its life. While most of these microorganisms pass through the insect gut’s lumen or interact with the host immune system, certain species are permitted to establish and proliferate within the gut environment called “commensal”. These commensal microbes provide metabolic assistance, secondary metabolite production, immune system support, pathogen clearance, gut homeostasis maintenance, intestinal tissue regeneration, and other valuable contributions to the host (43–45). In *Drosophila*, the gut microbiota encompasses 5-20 commensal bacteria species, mainly from the *Acetobacteraceae* and *Enterobacteriaceae* families or the *Lactobacillale* order, including *Lactobacillus plantarum, L. brevis, Acetobacter pomorum, Gluconobacter morbifer*, and *Commensalibacter intestine* (45). Although gut microbiota compositions vary due to fluctuations in environmental conditions and food availability, numerous studies have consistently identified the following dominant bacterial families in the *Drosophila’*s gut: *Acetobacteraceae* (55%), *Lactobacillaceae* (31%), *Leuconostocaceae* (4%), *Enterobacteriaceae* (3%), and *Enterococceae* (2%) (46). Furthermore, ingesting microorganisms, such as *Rhodiola crenulate*, enhances survival rates and induces antimicrobial peptide gene expressions in *D. melanogaster* following exposure to pathogens or toxic compounds (47).

Multiple studies have revealed that the gut microbiota influences host immune responses and various physiological activities that include larvae development, nutrient uptake, behavior, and environmental signal responses (48). The gut microbiota produces distinct metabolites sensed by host intestinal receptors, thereby triggering host metabolism (49). Interactions among microbes can influence triglyceride content, highlighting the microbiota’s influence on host metabolic processes (50). Notably, *Acetobacter persici* in the *Drosophila* gut prompts age-dependent metabolic purine level shifts by activating the innate immune signaling imd pathway in renal tubules (51). Furthermore, selective consumption of dietary components mediated by microbiota can modify the nutritional balance of food and ultimately impact the nutritional status of the host (52). Microbiota-induced signaling and regulatory networks involving genes associated with nutritional traits contribute to the host’s nutritional status (53). A substantial larvae accumulation in a specific area can alter food’s chemical and bacterial composition, effectuating growth time, pupation height, viability, and body mass alterations without affecting gut microbiota (54). The gut microbial community’s composition in insects depends on host-specific factors, with colonization transpiring in specific gut regions (55). Additionally, food sources can influence a microbiome’s establishment by reducing predominant member abundance and allowing new microorganisms to colonize (56). Introducing a single microorganism to the *D. melanogaster* gut can alter the microbial community’s symbiotic composition without exhibiting extensive antibacterial activity, potentially influenced by food sources. For example, *Rhodiola rosea* significantly increases *Acetobacter* abundance while diminishing *Lactobacillales*, engendering a symbiotic association (57).

*Drosophila* spp. exhibits bidirectional signaling through the “microbiota-gut-vagus-brain axis,” where gut microbiota can activate host neural circuitry and influence foraging behavior, stress-anxiety responses, and empathy development (58). During undernutrition, specific microbiomes, such as *L. plantarum*, induce the insulin/IGF-1 signaling (IIS) pathway, regulate glucose import and glycogen synthesis, and increase free amino acid levels by promoting gut peptidase expression via the imd pathway. A genetic *L. plantarum* screening identified an operon responsible for modifying bacterial cell walls, incorporating D-alanylated teichoic acids, which stimulate host enterocytes to induce gut peptidases (59). The gut microbiota, including *Acetobacter* spp. and yeast, also modifies host chemosensory responses, feeding preferences, and behavior, depending on the host-microbial history and identity. Different olfactory receptors and pathways are involved in sensing and responding to these changes, known as Olfactory-Guided Microbial Preferences by the host (60).

In addition, the *Drosophila* gut microbiota manages learning, memory, and sleep homeostasis aspects dependent on the host’s genetic background (61). Aggressive behaviors in both male and female *Drosophila* can be regulated by the microbiome, swaying mate selection during development (62). Interactions between *Drosophila* and its gut microbiome are foundations for developing new therapeutic drugs and understanding downstream consequences for host health through genome analysis. Notably, these findings have also extended to human precision medicine (63). Various factors influence the interplay between microbiota and their host concerning immune induction. The immune response in *D. melanogaster* incorporates both humoral and cellular components, including the production of antimicrobial peptides, ROS, reactive chlorine species (RCS), phagocytic cells, and melanization. The gut microbiota directly or indirectly aids in activating the immune response (64, 65). Among prevalent *D. melanogaster* microbiota*, Lactobacilli* spp. activate the NADPH Oxidases (NOX) pathway in gut tissue, releasing ROS and reducing microbiota overgrowth. Conversely, pathogenic bacteria stimulate ROS and RCS production through the dual oxidase system (Duox), which helps maintain host gut homeostasis (64, 65).

Microbiota and pathogens trigger the humoral immune response, inducing the production of antimicrobial peptides through imd and Toll regulatory pathways. These processes are initiated by PRRs in response to conserved microbial-associated molecular patterns (MAMPs) (64, 65). PGRPs, such as PGRP-LC, PGRP-LE, PGRP-SD, PGRP-LB, and PGRP-SC, within the IMD signaling cascade also recognize peptidoglycans from the microbiota. Notably, PGRP-LB has been found to protect the host from the harmful peptidoglycan effects on the hemolymph and other organ systems (64, 65). The EGFR and JAK-STAT signaling pathways are responsible for gut repair prompted by gut microbiota and pathogens, and the pathway activated depends on the damage sustained. Additionally, histone demethylase KDM5 deficiency begets an overactive IMD response and dysbiosis, whereas *L. plantarum* normalizes IMD activation and reduces dysbiosis (65). *Vibrio cholerae’*s Type 6 Secretion System (T6SS*)* and its interaction with *Acetobacter pasteurianus* microbiota induce the imd pathway in *D. melanogaster*, triggering immune responses (66). *L. plantarum*, a gut microbiota, can protect *D. melanogaster* from common pathogens, such as *Pseudomonas aeruginosa* and *Serratia marcescens*, by inducing the host’s immune response (56). Moreover, certain yeast commensals in *D. melanogaster* protect against the pathogenic fungus *Aspergillus flavus* (67). *L. plantarum* also mitigates *D. melanogaster’s* susceptibility to the pathogenic fungus *Diaporthe FY* (68). Furthermore, *Erwinia carotovora* pathogen infection and commensal microbes can trigger lipid utilization through enterocytes, but with distinct outcomes prevalent upon microbial cues, imd signaling, ROS production, and lipid utilization (64). Cell type-specific responses within the gut can also induce immune responses and are recognized by the gut microbiota. For instance, imd pathway activation in enterocytes invokes an immune response, activation in enteroendocrine cells promotes lipid utilization and improves anabolic growth (64). The microenvironment is also significant in maintaining constitutive immunity and gut microbiota concentration (69). Similarly, antimicrobial peptides and lysozymes, two immune effector families, regulate gut microbiota composition and abundance (70).

### 3.2 Gut microbiota in *T. molitor*

Like *Drosophila*, commensal microorganisms in *T. molitor* also affect gut immune responses and various physiological activities. The surrounding environment is a substantial source of diverse beneficial and pathogenic microorganisms. Different microorganism types could enter the *T. molitor* gut and establish themselves as symbionts or pathogens. Symbiotic microorganisms contribute to numerous physiological activities and environmentally friendly actions, such as detoxification in phytophagous insects, lignocellulose degradation in xylophagous insects, and antimicrobial compound production to protect against pathogens (71).

Experimental data has revealed that *T. molitor* harbors *Bacillus*, *Lactococcus, Weissella, Escherichia*, and *Clostridiaceae* spp. in an open environment but is dominated by *Enterococcus* spp. and some unclassified *Enterobacteriaceae* in a closed ecosystem. Microorganisms in the open environment actively contribute to host metabolism and immune activity, whereas the closed ecosystem offers the opportunity to select probiotic strains for improving growth efficiency (72). Another study on the gut of *T. molitor* identified fermentative bacteria, such as *Weissella* and *Lactococcus* spp.*;* proteolytic bacteria responsible for protein degradation, like *Rahnella* and *Cronobacter* spp.*; and* other microbiota groups, including *Lactobacillus, Bacillus, Spiroplasma, Clostridium, Enterobacter*, and *Pantoea* found in different concentrations across gut regions, with the hindgut containing the most diversity (73). Moreover, the gut microbiota in *T. molitor* was revealed to have significant plasticity in response to environmental factors, particularly soil. The composition of microbiota can rapidly fluctuate from one commensal group to another based on alterations in soil composition (74).

*T. molitor* and its gut microbiota degrade polystyrene (PS), polyvinyl chloride (PVC), polyethylene (PE), acrylonitrile butadiene styrene (ABS), polyurethane (PU), bio-based cross-linked polymers, and other plastic polymers (75). Research findings have identified eight specific microorganisms responsible for polystyrene biodegradation in *T. molitor*: *Citrobacter freundii, Klebsiella aerogenes, S. marcescens, Stenotrophomonas maltophilia, Bacillus thuringiensis, P. aeruginosa, Enterococcus faecalis*, and *Enterobacter asburiae.* It has been studied that *T. molitor* gut secretory factors and gut microbiota contribute to plastic degradation. Emulsifying factors with 30-100 kDa molecular weights were found in the *T. molitor* gut. In contrast, gut microbiome-produced secretory factors with molecular weights below 30 kDa were responsible for degrading plastic molecules (76). In addition, *T. molitor’s* gut microenvironments may be plays a crucial for plastic biodegradation. Therefore, combining *T. molitor*, specific gut microbiota, and an artificial microenvironment’s design will be a promising chamber for effective plastic degradation (76, 77).

Moreover, studies have demonstrated that although polystyrene provides some nutrients to *T. molitor*, but polystyrene has negative impacts on growth and development (78). *T. molitor* cannot survive on a diet consisting solely of plastic; additional essential nutrients are required (79). For example, different food supplements, such as bran with a 7:1 (w/w) plastic ratio, can promote *T. molitor* growth (80). Another study revealed that for polystyrene degradation, *Erwinia olea, Lactococcus lactis*, and *Lactococcus garviae* were the dominant microbiota in the gut of *T. molitor* (81). Similarly, for polyurethane degradation by *T. molitor’*s different gut microbiota, such as *Paraclostridium*, *Chryseobacterium, Kosakonia*, and *Pseudomonas*, were found to be responsible. During the degradation process, they can degrade the polymer by 35% within 17 days, but it experiences a weight loss of 14% (82). On the other hand, *T. molitor’s* gut microbiota breakdown long-chain and branched polymers by diffusing extracellular depolymerase, producing shorter chains containing 16 carbon atoms. The microbiota utilizes fatty acid degradation pathways to generate intermediate products during plastic degradation, while *T. molitor* acts as a downstream decomposer (79). Additionally, *T. molitor’s* gut microbiota can create cracks and holes in plastic sheets, form biofilms, and utilize them as a carbon source for growth (83). Furthermore, plastic molecular weight critically affects biodegradation. Plastic with low, medium, high, and ultra-high molecular weights can significantly influence metabolic pathways, induce intestinal dysbiosis, and alter gut microbiome composition (81, 84). Generally, *T. molitor* employs an extensive depolymerization process for plastic biodegradation, although the depolymerization of ultra-high molecular weight plastics is limited (84).

In addition, for plastic degradation, the gut microbiota involves the secretion of siderophores such as enterobactin and yersiniabactin, enzymes, and specific enzymatic genes such as undecaprenyl-phosphate galactose phosphotransferase [EC:2.7.8.6]. Furthermore, the supernatant of intestinal content acts as a surfactant, enhancing the hydrophilicity of plastic and providing physical support for the gut microbiomes (77). A fascinating discovery revealed that *Enterobacter hormaechei LG3* produces a biofilm on the plastic’s surface for degradation under anaerobic conditions. In this study, researchers identified degradative enzyme-expressing genes of the peroxidase family, including *tpx, ahpC*, and *bcp* (85). Another study demonstrated that phosphatases, esterases, leucine arylamidase, ß-galactosidase, ß-glucuronidase, α-glucosidase, ß-glucosidase, chitinase, α-mannosidase, α-fucosidase, and numerous other enzymes are involved in plastic degradation. Furthermore, the study suggested that enzymatic activity plays a more substantial role in plastic biodegradation than gut microbiota (86).

Moreover, to the environmental considerations regarding plastic degradation, *T. molitor* holds promise and has been approved by the European Union as a protein source to meet global food demand. Recent efforts have focused on enhancing *T. molitor’*s nutritional value by incorporating probiotic strains, such as *Pediococcus pentosacceus KVL-B19-01* and *Enterococcus faecium 669*, as commensal gut bacteria. These strains contribute as gut microbiota and promote host growth and development. Moreover, they protect against entomopathogenic fungi, such as *Metarhizium brunneum* (87). *P. pentosaceus* significantly protects *T. molitor* from six different insect pathogenic strains, including *B. thuringiensis* and species of *Serratia* and *Pseudomonas* bacteria (88). Another research study demonstrated that introducing *Lactobacillus* and *Bifidobacterium* strains, such as *Lactobacillus casei 01* and *Bifidobacterium animalis ssp*. *lactis Bb1* into the *T. molitor* gut, optimized them as gut microbiota without adversely affecting the host. In addition, these introduced strains can produce short-chain fatty acids (SCFA) and lactate (89).

*J. Poveda et al.* investigated *T. molitor* frass’ potentiality, including gut microbiota, as a biofertilizer and verified that it can enhance plant growth, even under stressful conditions (90). Conversely, parasitic symbionts hinder *T. molitor* mass production, as these symbionts can transfer to humans when *T. molitor* is used as a food source (91). When parasites are introduced into the insect gut microbiota, they stimulate the innate immune system through nonspecific cellular constituents known as hemocyte. Hemocytes respond to infectious agents by activating the phenoloxidase enzymatic cascade, ultimately producing specific antimicrobial peptides. However, parasites can alter the gut microbiota due to their transient nature, and in some cases, the host gut microbiota participates in establishing these parasites (92). Furthermore, *T. molitor* gut microbiota assists in detoxifying toxic food-derived compounds, such as plant-derived glucoside salicin. In summary, *T. molitor’s* immune mechanisms and gut microbiota are strikingly adaptable as vital host defense components (93).

## 4 *D. melanogaster’s* and *T. molitor*’s gut immune response

Diverse immune signaling pathways are intricately involved in mediating the gut immune response in *D. melanogaster* and *T. molitor.* These pathways are triggered by various signaling molecules released by pathogens and are sometimes associated with commensal microorganisms. The specific immune signaling pathways activated upon gut infection depend on the infecting microorganism’s nature and composition. The following sections explore the immune signaling pathways crucial for maintaining gut homeostasis and defense mechanisms.

### 4.1 Imd signaling pathway in *D. melanogaster*

**Figure 3:**
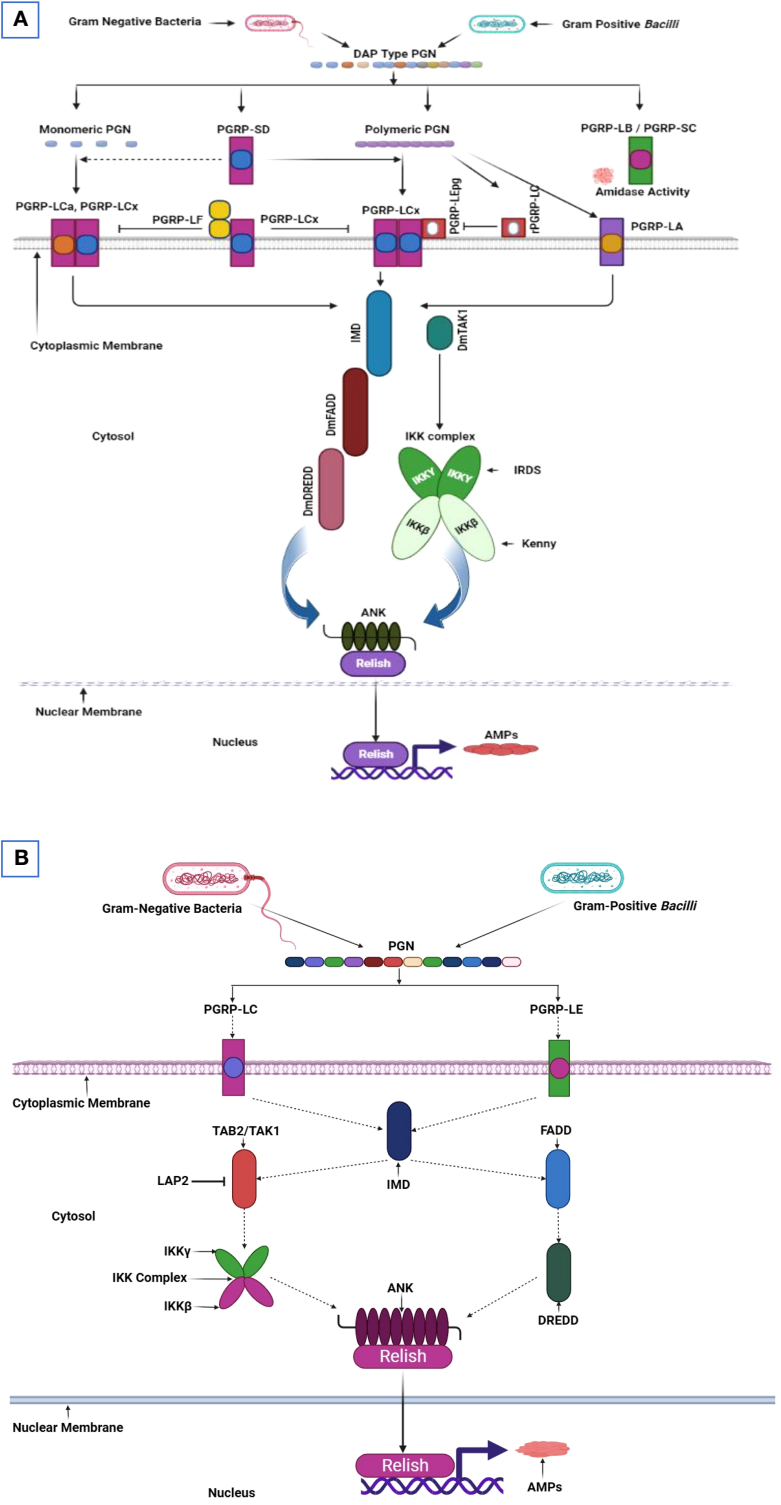
Imd pathway in *D. melanogaster* and *T. molitor*. **(A)** The immune deficiency (imd) pathway in the *Drosophila* midgut concurrently initiates in the proventriculus, anterior, median, and posterior regions. All receptors in these regions are essential for imd pathway activation. Peptidoglycan (PGN) recognition proteins (PGRP)-LEs and PGRP-LCs within the insect gut identify the polymeric and monomeric diaminopimelic acid (DAP) type PGN derived from gram-negative bacteria and gram-positive bacilli. These recognition signals are then translocated to the imd pathway within the cytoplasm. The intracellular domain of PGRP-LCs is subsequently activated and recruit IMD and, form a complex with the fas-associated protein and the death domain (dFADD*)*. This complex facilitates cleaving phosphorylated Relish, a protein with an N-terminal Rel DNA-binding domain and inhibitory ankyrin (ANK) repeats. The Relish domain is translocated to the nucleus, where it binds to NF-κB-response elements and begins transcribing genes encoding antimicrobial peptides. Meanwhile, the inhibitory Relish domain remains in the cytoplasm. Relish phosphorylation is mediated by a κB kinase (IKK) complex inhibitor, comprising immune responses deficient 5 (Ird5) and Kenny. This complex is activated by the mitogen-activated protein (MAP) kinase transforming growth factor (TGF)-β activated kinase1 (dTAK1), operating in an IMD and FADD-dependent manner. **(B)** The imd pathway activates in response to Gram-negative bacteria and Gram-positive bacilli infection in *T. molitor’*s gut. Signal transduction within gut cells hypothetically incorporates imd pathway activation, which comprises a death domain that facilitates *Tm*FADD (TAK1 activator) and *Tm*Dredd (a caspase) recruitment. The activated *Tm*TAK1, a protein kinase, then phosphorylates Relish through *Tm*IKK-β, -γ, and -ϵ. This phosphorylation event translocates the Rel domain into the nucleus, initiating antimicrobial peptide gene transcription. This illustration has been adopted from *M. A. M. Kojour et al* and *Y. Lu et al* (94, 95).

Peptidoglycan, a bacterial cell wall component derived from Gram-negative bacteria and Gram-positive bacilli, recognition mediates the immune response of gut cells in *D. melanogaster.* The peptidoglycan structure consists of 1,4-linked N-acetylglucosamine and N-acetylmuramic acid cross-linked sugar chains, four amino acids, and meso-diaminopimelic acid, forming what is known as DAP-type peptidoglycan. The detection of this peptidoglycan by *Drosophila* gut cells relies on the presence of PGRPs. Notably, the genes responsible for encoding PGRP are categorized by their transcript lengths. Upon DAP-type peptidoglycan binding, PGRP-LC and PGRP-LE transmembrane receptors activate the imd pathway (**Figure 3**). This binding event generates a signaling complex involving IMD, Dredd, and fas-associated protein and the death domain (FADD), subsequently translocating to the plasma membrane. Dredd is activated through K63-ubiquitination mediated by the E3-ligase inhibitor of apoptosis 2 (IAP2). Dredd cleaves IMD at its N-terminus, revealing an IAP2-binding motif and facilitating its interaction with IAP2’s BIR domain. As a result, the TAK1/TAB2 complex is recruited to the signaling complex. TAK1 then functions as a MAPKKK kinase and activates the IKK complex, which comprises the catalytic activity subunit IRD5 and the regulatory subunit “Kenny”. Next, the activated IKK complex phosphorylates Relish at multiple sites, activating various transcription factors, including RNA polymerase II. Negative imd pathway regulation incorporates the amidase activity of specific PGRPs, including PGRP-LB, PGRP-SC1a, PGRP-SC1b, and PGRP-SC2, to reduce bacterial peptidoglycan levels. Additional negative regulators include defense repressor 1 (Dnr1) to inhibit Dredd, Caspar for impeding Dredd-dependent Relish cleavage, Trabid at the TAK1 level, the deubiquitinating enzyme cylindromatosis (CYLD), SkpA (a subunit of the SCF E3 ubiquitin ligase targeting Relish), and transcriptional repressors that include caudal and the Oct1 homolog Nubbin (45, 96, 97).

The predominant intracellular receptor for monomeric peptidoglycan (PGN) in the *Drosophila* midgut is PGRP-LE. Conversely, in the proventriculus and hindgut, PGRP-LC binds to PGRP-LE in the ventriculus, facilitating the identification of monomeric and polymeric PGN structures. Commensal bacteria commonly present in the *Drosophila* gut, such as *Acetobacter* and *Lactobacilli* spp., harbor DAP-type PGNs. These bacteria maintain basal imd pathway activation and contribute to gut homeostasis (96). A comprehensive investigation concluded that the imd pathway is pivotal in governing antimicrobial peptide production and preserving barrier integrity within the gut. Remarkably, the coordinated imd and JNK signaling activities have been implicated in gut cell shedding regulation and controlled transcription factor GATAe expression, effectively controlling pathogen infections (98). Intriguingly, another experimental study revealed that microbial metabolic signals, specifically the short-chain fatty acid acetate, can induce imd pathway activation in enteroendocrine cells. This activation subsequently triggers endocrine peptide Tachykinin (Tk) upregulation, promoting larval development by influencing lipid metabolism and insulin signaling (99).

The imd pathway is a crucial immune signaling pathway in *D. melanogaster*, strictly regulated by various factors. Negative regulators, such as Pirk and PGRP-LB, balance bacterial infection and gut microbiota tolerance, while PGRP-LE acts as a sensor (100). In investigating the response to killed bacteria in *Drosophila*, specifically *Escherichia coli* and *Staphylococcus aureus*, gender-specific variations were observed due to differential receptor, signaling molecule, and protein expressions (101). Several common pathogenic microbes inhabit *Drosophila’s* intestinal tract, including *Pseudomonas entomophila, Vibrio cholerae, Candida albicans, Enterococcus faecalis*, and *Staphylococcus xylosus.* Certain pathogens, like *S. marcescens* and *P. aeruginosa*, can breach the intestinal barrier and activate the imd pathway (102, 103). While gram-positive bacteria generally activate the Toll pathway, a study demonstrated that the imd pathway and ROS partake in clearing ingested *S. aureus*, highlighting their versatile roles in insect gut immune defense (104). Microbial metabolites, such as acetate, can stimulate the imd pathway in anterior midgut enteroendocrine cells, producing antimicrobial peptides to protect against pathogens (105). In addition, Histone acetyltransferase Tip60 is an essential regulator in downregulating the imd signaling pathway by blocking dietary acetate and other acetyl-CoA sources, thereby blocking the pathway and fine-tuning gut immunity (106). On the other hand, the commensal gut microbiota can induce necrosis, activate the imd pathway, and alter bacterial populations, impacting *D. melanogaster’s* lifespan (107). Furthermore, the imd pathway and mustard mutants suppress *V. cholerae* infection and cholera toxins by inducing intestinal stem cell proliferation. This protective mechanism facilitates epithelial regeneration, protecting against pathogenic invasion (108). The imd signaling pathway is also vital for maintaining gut microbiota lysozyme concentrations, further contributing to gut homeostasis (70). The basic similarities and differences of imd and Toll signaling pathways in *D. melanogaster* and *T. molitor* have mentioned below in consideration of recognition receptor, adapter molecule, regulatory molecule, transcription factor and proteolytic cascades (**Table 1**). Experimental studies reveal that gut and Malpighian tubules of *D. melanogaster* are not directly related to the expression of genes related Toll signaling pathway. However, in consideration of *T. molitor* the gut immune response is stimulated by the Toll and imd immune signaling pathways (**Table 2**). The imd is the major regulatory pathway for gut and Malpighian tubules in *D. melanogaster.*

**Table 1:** Basic similarities and differences of imd and Toll signaling pathway in *D. melanogaster* and *T. molitor*.

imd Signaling Pathways			
Functions	*D. melanogaster*	*T. molitor*	References
Recognition receptors	PGRP-LCx PGRP-LCa PGRP-LE	PGRP-LC PGRP-LE	(94, 109, 110),
Adapter molecules	IMD FADD DREDD	IMD FADD DREDD	(94, 109),
Regulatory molecule	IKK	IKK	(109)
Transcription factor	Relish	Relish	(109)
Toll Signaling Pathways			
Functions	*D. melanogaster*	*T. molitor*	References
Recognition receptors	PGRP-SA PGRP-SD GNBP1 GNBP3	PGRP-SA PGRP-SD GNBP1 GNBP3	(94, 109),
Proteolytic cascades	ModSP Grass Sphinx1/2 Spirit Spheroide SPE Pro-spätzle Spätzle-1 Spätzle-2 Spätzle-3 Spätzle-4 Spätzle-5 Spätzle-6	MSP SAE SPE Pro-spätzle Spätzle-1b Spätzle-like Spätzle-3 Spätzle-4 Spätzle-5 Spätzle-6	(94, 109, 111–117)
Receptors	Toll-1 Toll-2 Toll-3 Toll-4 Toll-5 Toll-6 Toll-7 Toll-8 Toll-9 Toll-10	Toll-2 Toll-3 Toll-7	(118–121)
Regulatory molecules	MyD88 Tube Pelle Pellino	MyD88 Tube Pelle Pellino TRAF Cactin	(94, 122, 123),
Transcription factor	DIF Dorsal	DIF Dorsal	(94, 124),

**Table 2:** Tissue specific gene expression of imd and Toll pathway in *D. melanogaster* and *T. molitor*.

imd Signaling Pathway						
Tissue/Organs	*D. melanogaster*	Responsible Gene	References	*T. molitor*	Responsible Gene	References
Gut	Yes	*IMD/Relish*	(109, 125),	Yes	*PGRP-LE* *IKKγ* *Relish*	(109)
Malpighian tubules	yes	*Relish*	(126)	Yes	*IKKβ*	(127)
Fat bodies	Yes	*IMD/Relish*	(109)	Yes	*IKKγ* *IKKε* *Relish*	(109)
Whole body	Yes	*PGRP-LC*	(128)	Yes	*IMD*	(109)
Hemolymph	Yes	*IMD/Relish*	(109)	Yes	*IKKγ* *Relish*	(109)
Toll Signaling Pathway						
Tissue/Organs	*D. melanogaster*	Responsible Gene	References	*T. molitor*	Responsible Gene	References
Gut	No	“N/A”	(129)	Yes	*PGRP-SA* *Spätzle-4* *Spätzle-5* *Toll-7* *DorX2*	(111, 113, 124, 130, 131)
Malpighian tubules	No	“N/A”	(129)	Yes	*Spätzle-5* *Toll-7*	(111, 130),
Fat bodies	Yes	*Toll-1* *Toll-2* *Toll-4* *Toll-5* *Toll-6* *Toll-7* *Toll-8* *Toll-9*	(132)	Yes	*PGRP-SA* *Spätzle-1b* *Spätzle-4* *Spätzle-5* *Toll-7* *DorX2*	(111, 113, 124, 130, 131)
Whole body	Yes	*MyD88*	(129)	Yes	*Spätzle-like*	(130, 133, 134),
Hemolymph	Yes	*Toll-1* *Toll-2* *Toll-8*	(132)	Yes	*Spätzle-1b* *Spätzle-4* *Spätzle-5* *Toll-7* *DorX2*	(113, 124, 130, 135),

N/A, Not Applicable.

### 4.2 Imd signaling pathway *T. molitor*

The imd pathway in *T. molitor* is activated upon the recognition of pathogens by PRRs, such as PGRP-LC or PGRP LE. An intracellular signal is transmitted to the adaptor protein IMD. The IMD forms a signal complex with dFADD, K63, and caspase DREDD which induces IMD cleavage (**Figure 3**). Then, the K63-polyubiquitin molecule activates TAK1 and TAB2’s ubiquitin-binding domain. Relish phosphorylation and the subsequent translocation, which produce antimicrobial peptides for pathogen clearance, depend on the Imd signaling upstream receptors; PGRP LE/LC, relevant adaptor molecules; FADD, DREDD, TAB2, TAK1, and the IKK complex comprising *Tm*IKK- and (94). Moreover, IKK/NEMO as a non-catalytic regulatory subunit is pivotal in the NF-ĸB pathway signaling (136).

Specific immune responses in various *T. molitor* tissues, particularly the gut, are mediated by nine distinct AMP genes: *Tenecin1, Tenecin4, Attacin1a, Attacin1b, Attacin2, ColeoptericinA, ColeoptericinC, Defensin*, and *Defensin-like*. Notably, suppressing IMD expression during *E. coli* infection exacerbates mortality rates and downregulates these nine AMP genes. Similarly, *C. albicans* infection reduces five AMP gene expressions, namely *Tenecin2, Defensin-like, ColeoptericinA, Attacin1a*, and *Attacin2* (137, 138). A comprehensive scientific study has elucidated *Tm*PGRP-LE’s significance as a sensor for inducing AMP production via the Imd pathway in *T. monitor’s* gut. *Tm*PGRP-LE mRNA levels increase during *Listeria monocytogenes*, *E. coli*, and *C. albicans* infections, influencing *Tm*Relish, *Tm*DorX1, and *Tm*DorX2 expression levels in *T. molitor’s* gut (139, 140). Furthermore, *Tm*Relish’s mRNA expression is upregulated in the gut following gram-negative bacteria infection, such as *E. coli*, and downregulation of *Tm*Relish leads to repression of the nine AMP genes (141).

### 4.3 JAK/STAT signaling pathway in *D. melanogaster*

When *D. melanogaster* ingests gram-negative bacteria, the imd signaling pathway activates, producing various antimicrobial peptides. The JAK/STAT signaling pathway synthesizes antibacterial and Drosomycin-like peptides with antifungal properties, specifically within *D. melanogaster’s* anterior midgut (142, 143). Scientific investigations have demonstrated that the JAK/STAT, JNK, and DUOX pathways contribute to epithelial renewal through cell proliferation, ensuring gut homeostasis when faced with pathogenic infections. The JAK/STAT signaling pathway’s activation is facilitated by hop Tum-l or upd-3 expressions, encouraging gut renewal during infection, and may also be involved in non-infectious stages (143).

**Figure 4:**
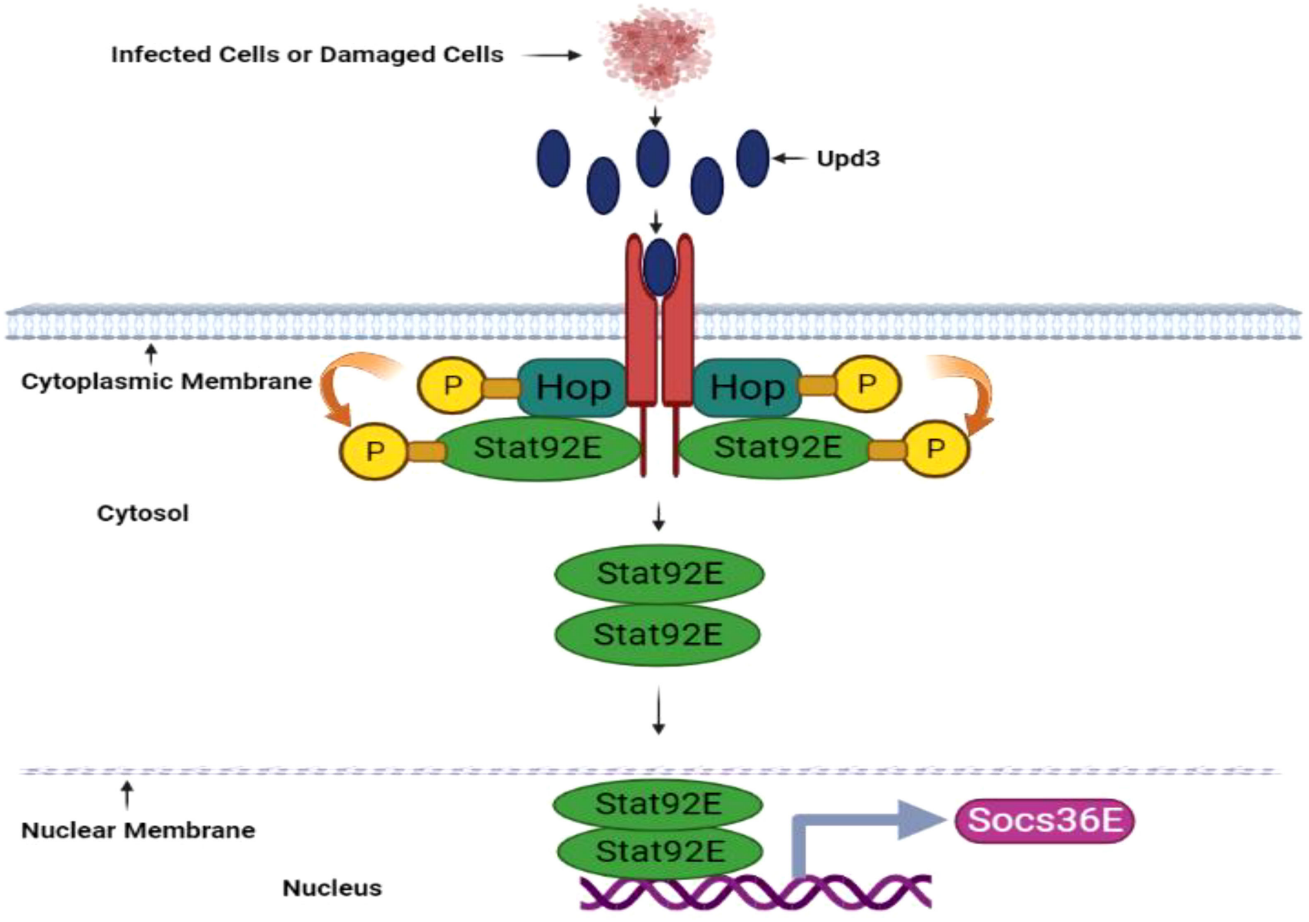
JAK/STAT pathway in *D. melanogaster*. The JAK/STAT pathway is activated upon pathogen invasion and produces antimicrobial peptides in the midgut, including antifungal Drosomycin-like peptides. This pathway is also vital for cell proliferation. Tissue damage prompted by pathogenic infection induces upd3 cytokine expression. Upon upd3 cytokine binding, the dimerized domeless receptors Hopscotch (Hop)/JAK activate, and Hopscotch phosphorylates itself and specific tyrosine residues on the receptors in cytoplasmic regions. These phosphorylated tyrosine residues serve as Stat92E binding sites, a transcription factor component of the STAT family. Hopscotch further phosphorylates Stat92E at tyrosine residues, promoting its dimerization and subsequent translocation into the nucleus. Once in the nucleus, Stat92E binds to the promoter regions of its target genes. The JAK/STAT signaling pathway also upregulates *Socs36E* gene expression This illustration has been adopted from *C. Kietz and A. Meinander et al* (144).

The JAK-STAT pathway, consisting of hopscotch/hop (JAK) and Stat92E (STAT transcription factor), in *D. melanogaster* responds to IL-3, IL-5, and IFN-γ hematopoietic cytokines and growth factors including granulocyte-macrophage colony-stimulating factor (GM-CSF), erythropoietin (EPO), growth hormone (GH), and prolactin. JAK activates when cytokines bind to their respective receptors, leading to receptor dimerization and subsequent association with the receptor’s cytoplasmic tail (**Figure 4**). Consequently, JAKs undergo phosphorylation at tyrosine residues, which serve as docking sites for STAT molecules (Stat92E)’s Src homology 2 (SH2) domains. Simultaneously, STAT is phosphorylated at tyrosine (Tyr-704) by JAKs, enabling STAT dimer formation and translocation into the nucleus. Subsequently, the STAT dimers bind to specific genes with the *TTCCCGGAA* binding sequence (108). Notably, a scientific study highlights the JAK-STAT pathway’s prominence in controlling intestinal stem cell differentiation, gut immunity, tissue regeneration, cell fate determination, and overall cell homeostasis in *D. melanogaster*. Additionally, *D. melanogaster* enteroendocrine cells secrete assorted neuropeptides responsible for regulating gut physiology and promoting growth (145).

### 4.4 Duox-ROS defense mechanism in *D. melanogaster*

**Figure 5:**
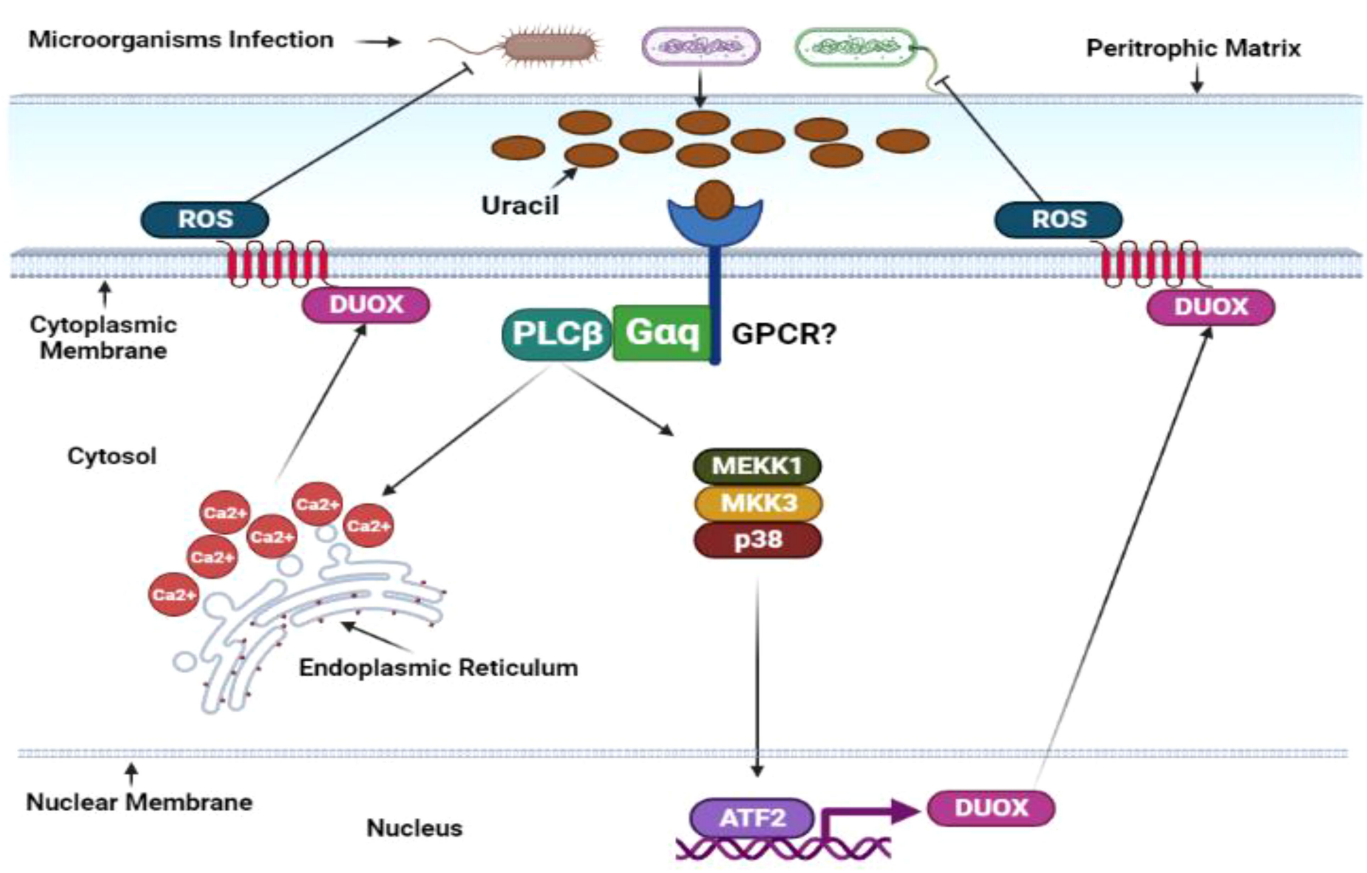
Duox-ROS defense mechanisms in *D. melanogaster*. The immune response in the insect gut produces reactive oxygen species. Similar to the IMD pathway, ROS is activated under basal conditions in response to gut microbiota and ingested microorganisms. However, this response is significantly amplified upon microbial infection. Microbially derived uracil triggers the activation of the adaptor molecules guanine-nucleotide-binding protein q subunit-α (Gαq) and phospholipase Cβ (PLCβ). These molecules stimulate inositol-3-phosphate synthesis, releasing intracellular calcium and transcribing Duox, the gene responsible for encoding oxidase enzymes. Duox transcription can also be induced by the p38 mitogen-activated protein kinase pathway and transcription factor Atf2. Under basal conditions, the activity of the Duox pathway is tightly regulated. The transcription of Duox is downregulated by MAPK phosphatase 3 (Mkp3), which is induced by PLCβ and Calcineurin B (CanB) in the absence of microbial infections. The specific activities of G protein-coupled receptors (GPCRs) in this pathway remain to be determined and require further investigation. This illustration has been adopted from *N. Buchon et al* (146).

A rapid reactive oxygen species burst is crucial for controlling bacterial growth in *D. melanogaster’s* gut. This ROS surge is facilitated by membrane-associated dual oxidase (Duox), a member of the nicotinamide adenine dinucleotide phosphate (NADPH) oxidase family (**Figure 5**). The p38, PGN-dependent, and PGN-independent pathways, which are integrated with the MEKK1-MEK3-p38-ATF2 pathway, regulate *DUOX* gene transcription (147). Certain gut-pathogenic bacteria, such as *Candida intestine, Vibrio, Klebsiella, Shigella, Pseudomonas*, and *Serratia*, secrete uracil, a nucleobase found in uridine. Moreover, DUOX activity is regulated by calcium ions released from the host gut cells’ endoplasmic reticulum, controlled by the inositol 1,4,5-triphosphate (IP3) receptor, phospholipase C-ß (PLCß), and G protein α-q (Gαq). Hence, researchers propose that DUOX-ROS activation transpires through the peptidoglycan-dependent pathway and a PGRP-LC/IMD/MEKK1/MKK3/p38/ATF2 cascade or the uracil-dependent PLCß pathway (45).

*Drosophila* gut cells’ intricacy includes the ability to discern commensal bacteria from their pathogenic counterparts by detecting uracil’s metabolic signature and secretion from pathogens during the stationary growth phase. Intriguingly, diverse phenotypic activities manifest among genetically similar strains of *Drosophila* gut epithelia. For example, while the *Lactobacillus brevis* strain EW exhibits colitogenic behavior, another *L. brevis* strain is a harmonious commensal bacterium. Uracil, notably integral to this intricate system, induces gut epithelium cell renewal through the DUOX signaling pathway and activates the JAK-STAT pathway via cytokine Unpaired-3 expression, orchestrating gut homeostasis (148). Uracil is formidably intricate, as it activates the Hedgehog (Hh) signaling pathway and spurs the cell adhesion molecule Cadherin 99C (Cad99C) into action. The resulting endosome formations are pivotal for activating PLCß/PKC/Ca2^+^-dependent DUOX machinery (149, 150). Furthermore, studies have unveiled the complex dance between lipogenesis induction, DUOX activity blockade, and ATG1-dependent lipophagy-induced lipolysis, a process indispensable for DUOX activation. TRAF3-AMPK/WTS-ATG1 pathway components deftly modulate infection-induced lipolysis, diminishing DUOX activation and any lingering *D. melanogaster* gut infections (151). Amidst intricacies, the Mesh protein regulates DUOX expression and gut bacteria proliferation by forming an Arrestin-mediated MAPK JNK/ERK phosphorylation cascade (152). In the grand tapestry of gut biology, DUOX-dependent reactive oxygen species are invaluable as they dictate the delicate balance between gut cell repair, pathogenic bacteria eradication, and host survival during infection (153).

### 4.5 JNK/FOXO signaling pathway in *D. melanogaster*

**Figure 6:**
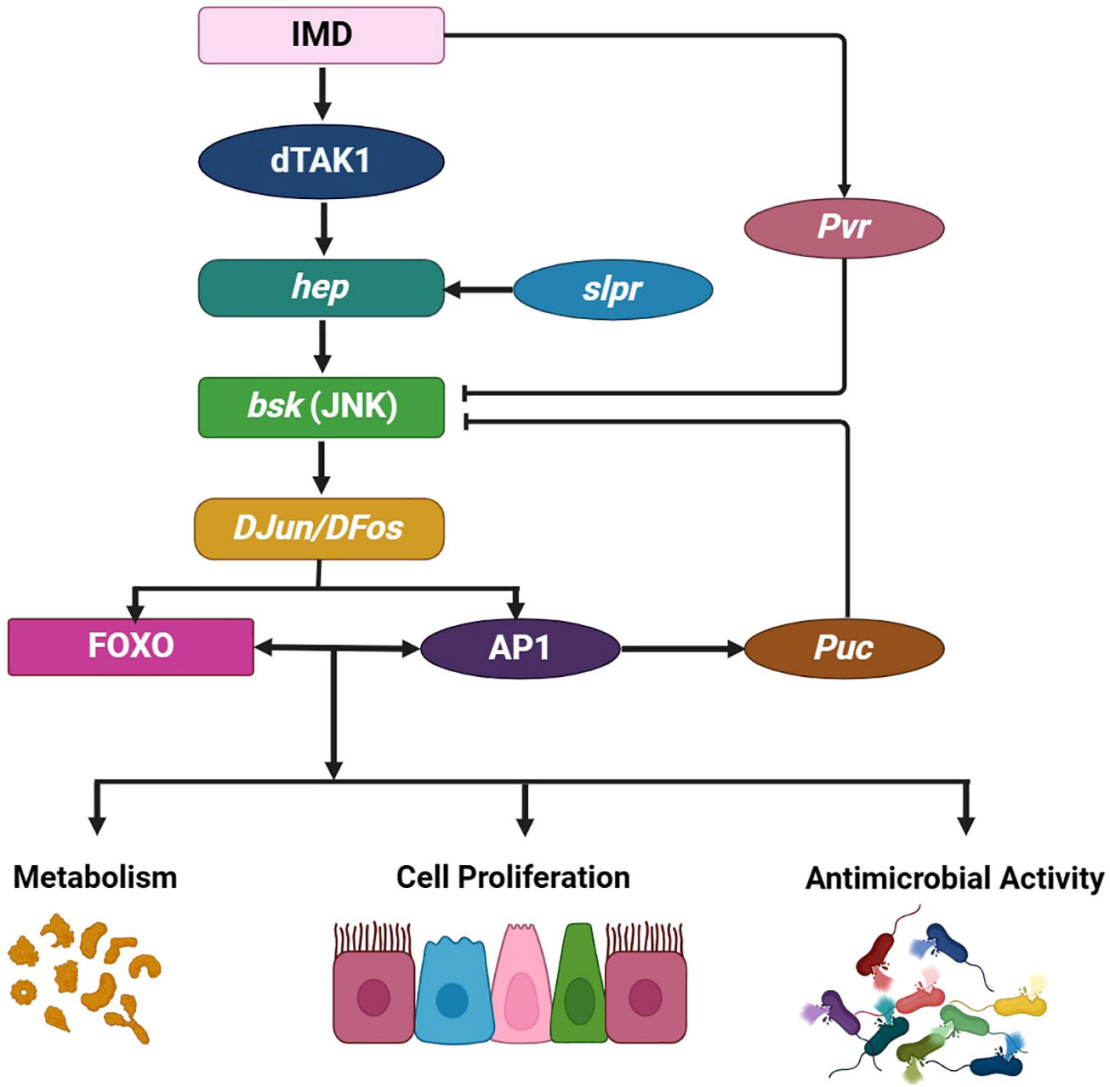
JNK pathway in *D. melanogaster*. The JNK pathway branches off from the Immune deficiency pathway at dTAK1, comprising the single gene basket (bsk) and two JNK kinases, Hemipterous (hep) and DJun/DFos. A broad range of intrinsic and extrinsic factors can activate this pathway. For example, the slipper (slpr) triggers JNK activation during embryonic dorsal closure, prompting various tissue- and context-specific cellular responses. The JNK signaling pathway is pivotal for regulating and promoting several crucial physiological processes that influence gut immunity and homeostasis, including antimicrobial responses regulated by the transcription factor Relish. This process also involves cytoprotection, which induces protective gene expressions, such as *hsp68, gstD1, fer1HCH*, and *mtnA*, in response to oxidative damage. Furthermore, the JNK pathway influences metabolism and controls inflammation-induced apoptosis, wound healing, and cell proliferation, which are regulated by Upd3. Negative JNK pathway regulation occurs through Puckered (Puc) and Pvr activity. This illustration has been adopted from *G. Tafesh-Edwards* and *I. Eleftherianos et al* (154).

Within the realm of cellular intricacies, the c-Jun N-terminal kinases, esteemed members of the mitogen-activated protein kinase family, stand as guardians of tissue homeostasis, diligently overseeing gut functionality, cell damage repair, the delicate dance between cell survival and death, the orchestrated responses to DNA damage, and regulate inflammatory cytokines. While *JNK1, JNK2*, and *JNK3* genes are responsible for JNK protein production in most organisms, a solitary basket (bsk)*-*encoded *JNK* gene and the two Hemipterous (hep) and DJun/DFos JNK kinases activate this multifaceted pathway in *D. melanogaster*. When the JNK signaling pathway diverges from the imd pathway, a distinct cascade at the dTAK1 stage begins (**Figure 6**). Traversing *D. melanogaster*’s embryonic stage, the JNK pathway relies on the slipper (slpr) for activation, setting transcription factors AP-1 into motion while simultaneously stimulating the Forkhead Box O transcription factor FOXO, thus maintaining gut homeostasis (155). Inquiries into dietary influences have revealed that high-sugar edibles incite gut stem cell differentiation, showcasing the JNK pathway’s complexity and prominence (156). Another study unveiled that activating the JNK/FOXO pathway stimulates dPrxV, essential for the subsequent pathogenic onslaught within *D. melanogaster’s* gut (157).

### 4.6 Other signaling pathways in *D. melanogaster*

Safeguarding against viral invasion in *D. melanogaster*’s gut epithelia is entrusted to the ERK signaling pathway. This intricate defense mechanism unfolds through two signals. First, the peptidoglycan recognition secretion factor Pvf2 emanates from the gut microbiota *A. pomorum*. The virus sets off a signaling cascade, triggering kinase Cdk9 release, which is indispensable for Pvf2 production (158). Further revelations have verified that the p38 pathway-mediated heat-shock factor is pivotal for safeguarding the gut against pathogenic assault by managing the JNK pathway (159).

## 5 *T. molitor’s* gut immune response

The pathways in *T. molitor*’s gut immune response are each directed by a diverse array of signaling molecules released by the gut and its commensal inhabitants. These immune signaling pathways vary by the specific invading microorganisms, unveiling a fascinating interplay between host and pathogen.

### 5.1 Toll signaling pathway

**Figure 7:**
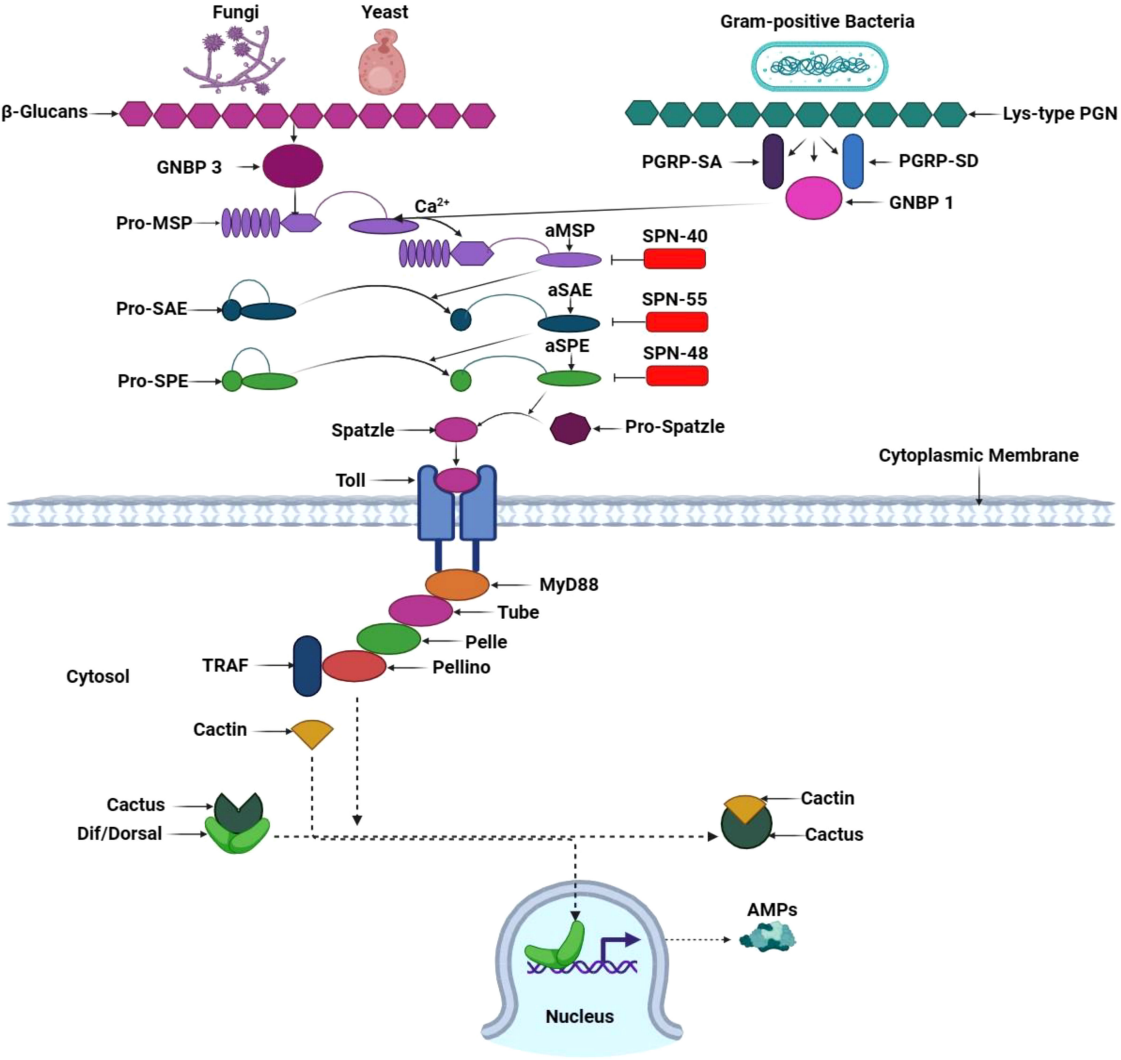
Toll signaling pathway in *T. molitor*. Circulating pathogen recognition receptor GNBP3 recognizes the fungal cell walls’ β-1,3 glucan component and activates the Toll pathway in *T. molitor*. Simultaneously, GNBP1 and the peptidoglycan recognition proteins PGRP-SA and PGRP-SD recognize Gram-positive bacteria’s lyse-type peptidoglycan (PGN). In *Tm*, PGN/β-1,3-glucan exposure binds Lys-type PGN to *Tm*PGRP-SA, thereby recruiting *Tm*GNBP1 and *Tm* modular serine protease (MSP) zymogen. In the presence of calcium ions (Ca2^+^), the PGN/*Tm*PGRP-SA/*Tm*GNBP1 complex induces the activation of the *Tm*MSP zymogen, resulting in the activation of *Tm*MSP. This initiates a proteolytic cascade involving three serine proteases: MSP, Spz processing enzyme (SPE), and SPE-activating enzyme (SAE). The activated SPE cleaves pro-Spz, leading to the generation of processed Spz. A specific three-serpin complex (SPN40, 55, and 48) combines with serine proteases to prevent pro-Spz processing and phenoloxidase-mediated melanin synthesis. Next, downstream signaling through the Toll pathway is activated when mature Spz binds to its receptor, triggering the *Tm*MyD88*, Tm*Tube*, Tm*Pelle*, Tm*Pellino, and *Tm*Tumor necrosis factor receptor-associated factor (TRAF) association in the cytosol. *Tm*Cactin binds to Cactus, resulting in the release of Dorsal-related immunity factor (Dif*)* and Dorsal from Cactus. This process translocates Dif and Dorsal into the nucleus, where they promote antimicrobial peptide gene transcription. This illustration has been adopted from *M. A. M. Kojour et al* and *K. Ryu et al* (94, 160).

The Toll signaling pathway begins when GNBP3 recognizes fungal ß-glucan and Lys-type PGN, namely PGRP-SA and GNBP1, from gram-positive bacteria. These extracellular PRRs activate a serine cascade, culminating in extracellular cytokine Spätzle induction and its binding with the Toll receptor ligand. Within this intricate network, the trimeric complex formed by adaptor protein MyD88, Tube, and the kinase Pelle invoke a series of phosphorylation events. Pelle phosphorylates Cactus, an inhibitory protein. Meanwhile, the transcription factors Dorsal and Dorsal-related immunity factor (Dif) within the cytoplasm traverse into the nucleus, where they generate an array of antimicrobial peptides (**Figure 7**) (161). Remarkably, the Toll signaling pathway’s activation is not initiated through direct contact with the microorganisms but rather upon detecting the cytokine-like polypeptide Spätzle, evidencing *Semmelweis* and *Osiris* genes’ influence in gram-positive-dependent Toll activation (94).

Regarding *T. molitor*’s gut immunity, *Tm*PGRP-SA mRNA levels are upregulated in response to *E. coli* and *C. albicans* invasion. This upregulation triggers antimicrobial peptide transcription, including *TmTenecin-2, -4; TmDefensin-2; TmColeoptericin-1, -2; and TmAttacin-1a, 1b, and -2* (139). The cystine knot protein *Tm*Spz-like is expressed upon *E. coli* invasion, generating numerous AMPs to protect the gut epithelia (162). Through a study on *Coleoptericin* gene expression in *T. molitor*’s gut immunity among *E. coli, S. aureus*, and *C. albicans;* it was discovered that the infection of *E. coli* tantalized the genes *TmCole B* and *TmCole C* of *Coleoptericin*, while fungi did not share the same allure (163). In addition, *TmToll-2* expression was elevated in young larvae gut after *E. coli, S. aureus*, and *C. albicans* invasion (164). *C. albicans* infection in young *T. molitor* larvae induced *TmToll-3* within the gut; however, AMP gene transcription factors were downregulated by *E. coli* infection (165).

## 6 Insect gut post-infection recovery

The potent production and proliferation of specialized daughter cells from intestinal stem cells are crucial in gut cell self-renewal following infection (136). ISC proliferation generates two distinct daughter cell types: ISC with self-renewal capacity and enteroblast cells capable of differentiating into enterocytes or enteroendocrine cells. Controlling ISC proliferation is paramount during gut epithelial damage from pathogens or toxic substances. Rapid ISC proliferation excessively accumulates cells, exhibiting pathogenic behavior. Conversely, if ISC proliferation is slowed, damaged cells cannot be adequately replaced, resulting in compromised gut integrity and ultimately leading to the demise of the host (166).

**Figure 8:**
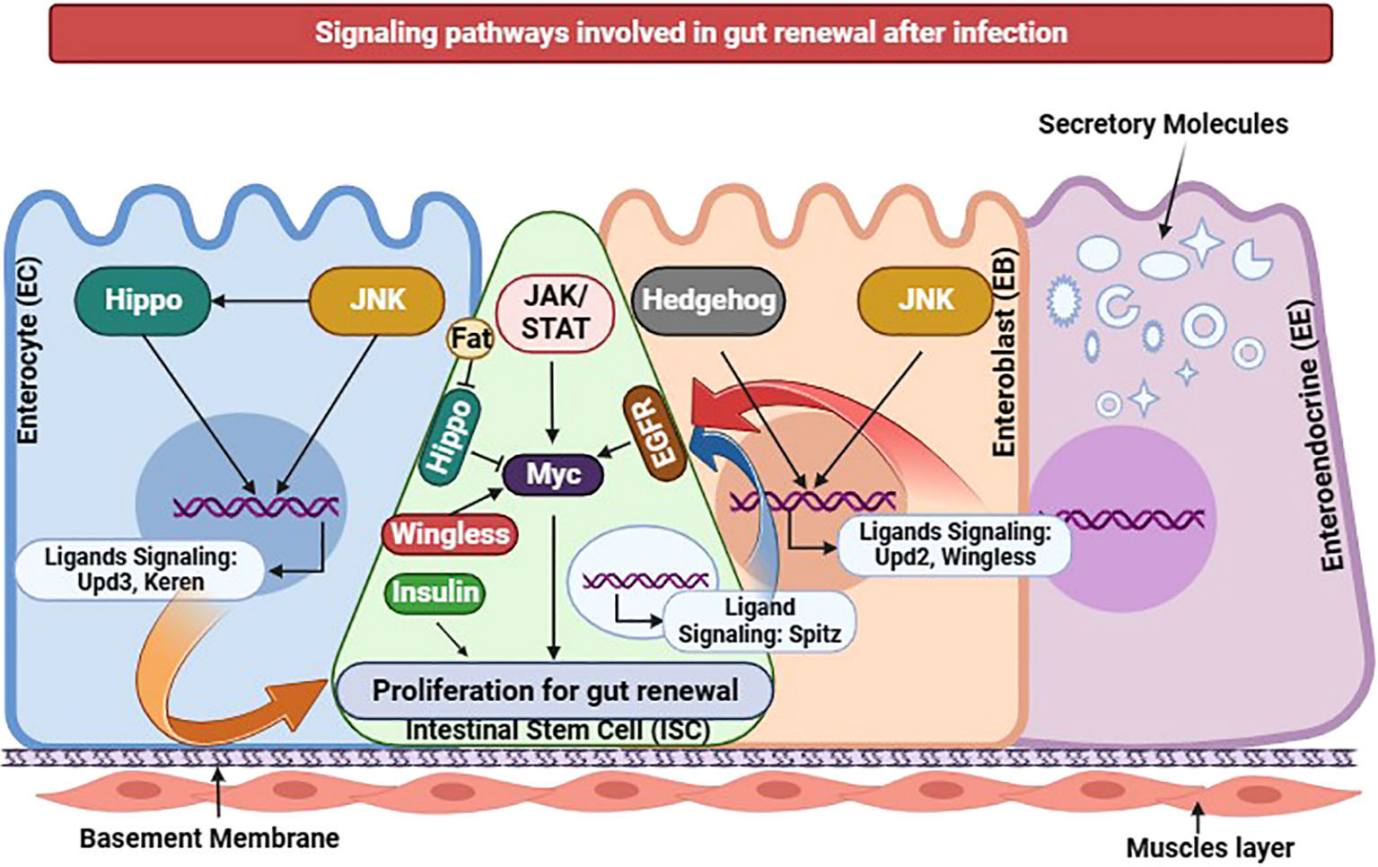
Insect gut renewal through different signaling pathways during infection in *D. melanogaster*. Various signaling pathways have been recently identified as regulators for intestinal stem cell proliferation. Jak/Stat, EGFR, Hippo, JNK, or Wingless pathway activation in ISCs alone can induce ISC proliferation. The transcription factor Myc functions as a downstream effector shared by Jak/Stat, EGFR, Hippo, and Wingless pathways in promoting ISC proliferation. Additionally, proper insulin receptor signaling in ISCs is necessary for its proliferation. In response to cellular stress, Enterocytes exhibit changes in gene expression regulated by the JNK and Hippo pathways, resulting in the induction of Jak/Stat pathway ligands, particularly Upd3, and EGFR pathway ligands, particularly Keren. Ligands of the EGFR pathway namely Vein and Spitz are expressed in visceral muscles and progenitors, respectively. Under stressed conditions, Enteroblasts produce Upd2 through activation of the Hedgehog pathway. EBs also express the Wingless ligand, regulated by the JNK pathway. The activity of the Hippo pathway in ISCs may be modulated by the intercellular interaction between two atypical cadherins: Fat in ISCs and Dachsous in ECs. Collectively, the diverse cellular components within the gut epithelium possess the ability to regulate ISC proliferation, functioning as intricate sensors that respond to environmental damage and initiate the gut renewal program. This illustration has been adopted from *J.-H. Lee et al* (167).

Moreover, a multitude of signaling pathways, namely JNK, JAK/Stat, Hippo, EGFR, Wg, Hh, and Dpp/BMP, are intricately involved in the complex process of gut recovery in insect (136). The regulatory molecule Myc influences downstream events within the Wingless, EGFR, Jak-Stat, and Hippo pathways, effectively proliferating intestinal stem cells (**Figure 8**). Conversely, in conditions of cellular stress within enterocytes the Upd3 ligands of the Jak-Stat pathway exhibit the ability to activate the JNK pathway, whereas the Keren ligands of the EGFR pathway can induce the activation of the Hippo pathway. When enteroblasts are stressed, Upd2 ligand production activates the Hedgehog pathway, and EBs activate Wingless ligands to stimulate the JNK pathway. Consequently, every cell within the gut possesses the remarkable capacity to regulate ISC proliferation; thus, each type is crucial in restorative processes following gut infection (166).

Nonetheless, researchers are actively investigating the interplay between intestinal renewal and immune pathways. While the imd pathway does not directly induce intestinal stem cell division, it does modulate commensal microorganisms within the gut to ensure homeostasis (168). Conversely, reactive oxygen species molecules, including SDS, DSS, and bleomycin, can stimulate ISC proliferation through the DUOX pathway. Similarly, ROS molecules can activate the Jak-Stat pathway by inducing redox-sensitive protein tyrosine phosphatases, the JNK pathway through thioredoxin, and the Wnt pathway via nucleoredoxin (166). One scientific study demonstrated that translational blockage triggered by *P. entomophila* infection subsequently released pore-forming toxins, and the host response producing reactive oxygen species can hinder immune response and impair gut repair. This impairment transpires through GCN2 kinase activation and rapamycin pathway inhibition (169). In response to apoptosis, pathogenic infections, and JNK-mediated stress signaling, enterocytes produce various cytokines, such as Upd, Upd2, and Upd3. These cytokines activate Jak/Stat signaling in ISC, promoting their proliferation and stimulating Delta/Notch signaling to facilitate gut tissue repair (170). In addition, the epidermal growth factor receptor (EGFR) pathway is initiated by Spitz, Keren, and Vein EGF ligands. Combined with the JAK/STAT pathway, this effectively triggers intestinal stem cell proliferation, facilitating proper infected gut tissue renewal from pathogen-induced damage (171).

Another investigation uncovered EGFR/MAPK pathway induction by the Insulin/Pi3K/TOR signaling cascade, serving as a crucial mechanism for gut renewal under stressful conditions by promoting the growth of EBs and ECs. The E2f1 transcription factor is essential for this induction; the Ras/Raf signaling pathway actively upregulates E2f1 levels and ensures proper transcriptional induction and fine-tuning of the EGFR/RAS/MAPK pathway activity, ultimately driving efficient gut regeneration (172). Moreover, during pathogenic infections within the gut, the YKi pro-growth transcription factor activates the Hippo pathway, influencing ISC differentiation and contributing to gut defense and repair (173). In a comprehensive gene expression study, 1833 genes were identified as responsible for ISC expression, 233 genes governed enterocyte function, 433 genes regulated enteroendocrine cell activity, and 2646 genes influenced Enteroblast behavior. Either directly or indirectly, these genes are vital for intricate gut tissue damage repair (174).

## 7 Conclusions and prospects

Despite these insights and explanations, numerous questions pertaining to insect gut immunity defense mechanisms of gut immunity in insects remain unanswered. Notably, inquiries arise regarding the mechanisms by which the immune system controls the composition and abundance of gut microorganisms. Additionally, the intricate relationship between gut microbiota and host behavior and physiological activities warrants further exploration. Furthermore, elucidating the precise mechanisms underlying gut microbiota dysbiosis remains a critical area of investigation. The differential activation of the Toll pathway in gut immunity between *D. melanogaster* and *T. molitor* raises intriguing questions, necessitating an examination of the underlying reasons for this discrepancy study. Moreover, unraveling the exact mechanisms that trigger the activation of intestinal renewal pathways in *T. molitor* presents an intriguing avenue for future research. Furthermore, understanding the intricate mechanisms underlying gut immunity during both pathogenic and non-pathogenic viral infections in *T. molitor* is of paramount importance. Exploration of the stages of infection, the duration required for gut tissue recovery following pathogen incorporation, and the precise pathogenic dose or number of infectious doses required for infection in *D. melanogaster* and *T. molitor* are crucial aspects that demand further investigation.

In the context of considering the utilization of *T. molitor* as a viable food source for human consumption, it is important to investigate the intricate interplay between the pathogens harbored within the *T. molitor* gut and the commensal microorganisms residing within the human intestinal ecosystem. A critical inquiry arises as to whether the commensal microorganisms present in *T. molitor* confer any advantageous attributes upon their introduction into the human intestinal milieu. Furthermore, during gut infections, *T. molitor* elicits the production of antimicrobial peptides as a defense mechanism against invading pathogens. An intriguing avenue for exploration emerges regarding the potential implications of these AMPs on human intestinal immunity. Could these AMPs be harnessed as a means to fortify human immune responses, potentially through targeted injections or other innovative modalities? Moreover, as *T. molitor* is ingested as a food source, various metabolites derived from diverse metabolic pathways are generated. It is imperative to ascertain the potential repercussions of these intermediate products on the integrity and functionality of the human intestinal tract. Meticulous investigations encompassing these concerns are promising for unraveling the intricacies underlying insect gut immune defense. Furthermore, such endeavors bear the potential for far-reaching applications spanning a vast array of disciplines.

## Author contributions

YH: Conceptualization, Funding acquisition, Supervision, Writing – original draft, Writing – review & editing. SA: Conceptualization, Investigation, Visualization, Writing – original draft, Writing – review & editing. MA: Conceptualization, Validation, Writing – review & editing.

## Glossary

**Table:**

PRRs	Pattern recognition receptors
AMPs	Antimicrobial peptides
imd	Immune deficiency
NF-kB	Nuclear Factor-kappaB
Duox	Dualoxidase
ROS	Reactive oxygen species
PPO	Prophenoloxidase
JAK/STAT	Janus kinase
WNT	Wingless and Int-1
ISC	Intestinal stem cell
EGFR	Epidermal Growth Factor Receptor
EECs	Enterocytes (ECs) Enteroendocrine cells
EBs	Enteroblasts
PM	Peritrophic membrane
ChtBD2	Chitin-binding domains
PMP	PM proteins
HPx1	Heme-dependent peroxidase 1
JcDV	Junonia coenia densovirus
Mf-mucins	mucus-forming mucins
Insulin/IGF-1	signaling (IIS) pathway
RCS	Reactive chlorine species
NOX	NADPH Oxidases
MAMPs	Microbial-associated molecular patterns
PGRPs	Peptidoglycan receptor proteins
T6SS	Type 6 Secretion System
PS	Polystyrene
PVC	Polyvinyl chloride
PE	Polyethylene
ABS	Acrylonitrile butadiene styrene
PU	Polyurethane
SCFA	Short-chain fatty acids
IAP2	Inhibitor of apoptosis 2
Dnr1	Defense repressor 1
CYLD	Cylindromatosis
DAP	Diaminopimelic acid
dFADD	fas-associated protein and the death domain
ANK	Ankyrin
IKK	Inhibitor κB kinase
Ird5	Immune responses deficient 5
TGF	Transforming growth factor
dTAK1	Activated *kinase1*
Hop	Hopscotch
JAK	Hopscotch/hop
Stat92E	STAT transcription factor
GM-CSF	Granulocyte-macrophage colony-stimulating factor
EPO	Erythropoietin
GH	Growth hormone
SH2	Src homology 2
Tyr-704	Tyrosine
NADPH	Nicotinamide adenine dinucleotide phosphate
IP3	Inositol 1
4	5-triphosphate
PLCß	Phospholipase C-ß
Gαq	G protein α-q
Hh	Hedgehog
Cad99C	Cadherin 99C
CanB	Calcineurin B
MAPK	Mitogen-activated protein kinase
Mkp3	MAPK phosphatase 3
GPCRs	G protein-coupled receptors
JNKs	c-Jun N-terminal kinases
bsk	Basket
hep	Hemipterous
slpr	Slipper
Puc	Puckered
GNBP3	Gram-negative binding protein 3
PGN	Peptidoglycan
Dif	Dorsal-related immunity factor
*Dm*	*Drosophila melanogaster*
*Tm*	*Tenebrio molitor*
MSP	Modular serine protease
SPE	Spz processing enzyme
SAE	SPE-activating enzyme
TRAF	*Tm*Tumor necrosis factor receptor-associated factor
DS	Dachsous

## References

[1] Miguel-Aliaga I Jasper H Lemaitre B. Anatomy and physiology of the digestive tract of drosophila melanogaster. Genetics (2018) 210:357–96. doi: 10.1534/genetics.118.300224 PMC6216580 30287514

[2] Zeng T Jaffar S Xu Y Qi Y. The intestinal immune defense system in insects. Int J Mol Sci (2022). doi: 10.3390/ijms232315132 PMC9740067 36499457

[3] Kuraishi T Binggeli O Opota O Buchon N Lemaitre B. Genetic evidence for a protective role of the peritrophic matrix against intestinal bacterial infection in Drosophila melanogaster. Proc Natl Acad Sci United States America (2011) 108:15966–71. doi: 10.1073/pnas.1105994108 PMC3179054 21896728

[4] Syed ZA Härd T Uv A van Dijk-Härd IF. A potential role for Drosophila mucins in development and physiology. PloS One (2008) 3:e3041. doi: 10.1371/journal.pone.0003041 18725942 PMC2515642

[5] Simões ML Gonçalves L Silveira H. Hemozoin activates the innate immune system and reduces Plasmodium berghei infection in Anopheles Gambiae. Parasites Vectors (2015) 8:12. doi: 10.1186/s13071-014-0619-y 25573379 PMC4297457

[6] Jang S Mergaert P Ohbayashi T Ishigami K Shigenobu S Itoh H. Dual oxidase enables insect gut symbiosis by mediating respiratory network formation. Proc Natl Acad Sci United States America (2021) 118. doi: 10.1073/pnas.2020922118 PMC7958442 33649233

[7] Lu A Zhang Q Zhang J Yang B Wu K Xie W. Insect prophenoloxidase: the view beyond immunity. Front Physiol (2014) 5:252. doi: 10.3389/fphys.2014.00252 25071597 PMC4092376

[8] Bang IS. JAK/STAT signaling in insect innate immunity. Entomological Res (2019) 49:339–53. doi: 10.1111/1748-5967.12384

[9] Zhang X Zhang F Lu X. Diversity and functional roles of the gut microbiota in lepidopteran insects. Microorganisms (2022) 10. doi: 10.3390/microorganisms10061234 PMC9231115 35744751

[10] Duplais C Sarou-Kanian V Massiot D Hassan A Perrone B Estevez Y. Gut bacteria are essential for normal cuticle development in herbivorous turtle ants. Nat Commun (2021) 12:676. doi: 10.1038/s41467-021-21065-y 33514729 PMC7846594

[11] Bai S Yao Z Raza MF Cai Z Zhang H. Regulatory mechanisms of microbial homeostasis in insect gut. Insect Sci (2021) 28:286–301. doi: 10.1111/1744-7917.12868 32888254

[12] Napoleão TH Albuquerque LP Santos ND Nova IC Lima TA Paiva PM. Insect midgut structures and molecules as targets of plant-derived protease inhibitors and lectins. Pest Manage Sci (2019) 75:1212–22. doi: 10.1002/ps.5233 30306668

[13] Ma E Zhu Y Liu Z Wei T Wang P Cheng G. Interaction of viruses with the insect intestine. Annu Rev Virol (2021) 8:115–31. doi: 10.1146/annurev-virology-091919-100543 33872516

[14] Smith CC Srygley RB Healy F Swaminath K Mueller UG. Spatial structure of the mormon cricket gut microbiome and its predicted contribution to nutrition and immune function. Front Microbiol (2017) 8:801. doi: 10.3389/fmicb.2017.00801 28553263 PMC5427142

[15] Subta P Yodsuwan P Yongsawas R In-On A Warrit N Panha S. Bacterial communities in three parts of intestinal tracts of carpenter bees (Xylocopa tenuiscapa). Insects (2020). doi: 10.3390/insects11080497 PMC7469164 32756386

[16] Chew YM Lye S Md. Salleh M Yahya A. 16S rRNA metagenomic analysis of the symbiotic community structures of bacteria in foregut, midgut, and hindgut of the wood-feeding termite Bulbitermes sp. Symbiosis (2018) 76:187–97. doi: 10.1007/s13199-018-0544-5

[17] Sharma P Sharma S Maurya RK Das De T Thomas T Lata S. Salivary glands harbor more diverse microbial communities than gut in Anopheles culicifacies. Parasites Vectors (2014) 7:235. doi: 10.1186/1756-3305-7-235 24886293 PMC4062515

[18] Lanan MC Rodrigues PA Agellon A Jansma P Wheeler DE. A bacterial filter protects and structures the gut microbiome of an insect. ISME J (2016) 10:1866–76. doi: 10.1038/ismej.2015.264 PMC5029173 26872040

[19] Abou El Asrar R Cools D Vanden Broeck J. Role of peptide hormones in insect gut physiology. Curr Opin Insect Sci (2020) 41:71–8. doi: 10.1016/j.cois.2020.07.004 32814267

[20] Singh SR Zeng X Zheng Z Hou SX. The adult Drosophila gastric and stomach organs are maintained by a multipotent stem cell pool at the foregut/midgut junction in the cardia (proventriculus). Cell Cycle (Georgetown Tex.) (2011) 10:1109–20. doi: 10.4161/cc.10.7.14830 PMC3100886 21403464

[21] Roelfstra L Vlimant M Betschart B Pfister K Diehl PA. Light and electron microscopy studies of the midgut and salivary glands of second and third instars of the horse stomach bot, Gasterophilus intestinalis. Med veterinary entomology (2010) 24:236–49. doi: 10.1111/j.1365-2915.2010.00881.x 20534009

[22] Bonelli M Bruno D Caccia S Sgambetterra G Cappellozza S Jucker C. Structural and functional characterization of hermetia illucens larval midgut. Front Physiol (2019) 10:204. doi: 10.3389/fphys.2019.00204 30906266 PMC6418021

[23] Buchon N Osman D David FP Fang HY Boquete JP Deplancke B. Morphological and molecular characterization of adult midgut compartmentalization in Drosophila. Cell Rep (2013) 3:1725–38. doi: 10.1016/j.celrep.2013.04.001 23643535

[24] Tokuda G Watanabe H Hojo M Fujita A Makiya H Miyagi M. Cellulolytic environment in the midgut of the wood-feeding higher termite Nasutitermes takasagoensis. J Insect Physiol (2012) 58:147–54. doi: 10.1016/j.jinsphys.2011.10.012 22085675

[25] Shi X Liu X Silver K Zhu KY Zhang J. Lethal giant larvae gene is required for normal nymphal development and midgut morphogenesis in Locusta migratoria. Insect Sci (2022) 29:1017–29. doi: 10.1111/1744-7917.12996 34978756

[26] Jiang H Edgar BA. Intestinal stem cells in the adult Drosophila midgut. Exp Cell Res (2011) 317:2780–8. doi: 10.1016/j.yexcr.2011.07.020 PMC6141237 21856297

[27] Shao Q Yang B Xu Q Li X Lu Z Wang C. Hindgut innate immunity and regulation of fecal microbiota through melanization in insects. J Biol Chem (2012) 287:14270–9. doi: 10.1074/jbc.M112.354548 PMC3340165 22375003

[28] Kuraishi T Hori A Kurata S. Host-microbe interactions in the gut of Drosophila melanogaster. Front Physiol (2013) 4:375. doi: 10.3389/fphys.2013.00375 24381562 PMC3865371

[29] Merzendorfer H Kelkenberg M Muthukrishnan S. Peritrophic matrices. In: Extracellular composite matrices in arthropods (2016). p. 255–324. doi: 10.1007/978-3-319-40740-1_8pp

[30] Rodgers FH Gendrin M Wyer CAS Christophides GK. Microbiota-induced peritrophic matrix regulates midgut homeostasis and prevents systemic infection of malaria vector mosquitoes. PloS Pathog (2017) 13:e1006391. doi: 10.1371/journal.ppat.1006391 28545061 PMC5448818

[31] Talyuli OAC Oliveira JHM Bottino-Rojas V Silveira GO Alvarenga PH Barletta ABF. The Aedes aEgypti peritrophic matrix controls arbovirus vector competence through HPx1, a heme-induced peroxidase. PloS Pathog (2023) 19:e1011149. doi: 10.1371/journal.ppat.1011149 36780872 PMC9956595

[32] Pigeyre L Schatz M Ravallec M Gasmi L Nègre N Clouet C. Interaction of a densovirus with glycans of the peritrophic matrix mediates oral infection of the lepidopteran pest spodoptera frugiperda. Viruses (2019) 11. doi: 10.3390/v11090870 PMC6783882 31533310

[33] Hegedus DD Toprak U Erlandson M. Peritrophic matrix formation. J Insect Physiol (2019) 117:103898. doi: 10.1016/j.jinsphys.2019.103898 31211963

[34] Wu K Yang B Huang W Dobens L Song H Ling E. Gut immunity in Lepidopteran insects. Dev Comp Immunol (2016) 64:65–74. doi: 10.1016/j.dci.2016.02.010 26872544

[35] Dias RO Cardoso C Pimentel AC Damasceno TF Ferreira C Terra WR. The roles of mucus-forming mucins, peritrophins and peritrophins with mucin domains in the insect midgut. Insect Mol Biol (2018) 27:46–60. doi: 10.1111/imb.12340 28833767

[36] Syed ZA Zhang L Ten Hagen KG. *In vivo* models of mucin biosynthesis and function. Advanced Drug delivery Rev (2022) 184:114182. doi: 10.1016/j.addr.2022.114182 PMC9068269 35278522

[37] Moriyama M Hayashi T Fukatsu T. A mucin protein predominantly expressed in the female-specific symbiotic organ of the stinkbug Plautia stali. Sci Rep (2022) 12:7782. doi: 10.1038/s41598-022-11895-1 35546182 PMC9095716

[38] Fuzita FJ Palmisano G Pimenta DC Terra WR Ferreira C. A proteomic approach to identify digestive enzymes, their exocytic and microapocrine secretory routes and their compartmentalization in the midgut of Spodoptera frugiperda. Comp Biochem Physiol Biochem Mol Biol (2022) 257:110670. doi: 10.1016/j.cbpb.2021.110670 34438074

[39] Terra WR Barroso IG Dias RO Ferreira C. Molecular physiology of insect midgut. (2019). pp. 117–63. doi: 10.1016/bs.aiip.2019.01.004pp.

[40] Jasper H. Intestinal stem cell aging: origins and interventions. Annu Rev Physiol (2020) 82:203–26. doi: 10.1146/annurev-physiol-021119-034359 31610128

[41] Funk MC Zhou J Boutros M. Ageing, metabolism and the intestine. EMBO Rep (2020) 21:e50047. doi: 10.15252/embr.202050047 32567155 PMC7332987

[42] Liu X Hodgson JJ Buchon N. Drosophila as a model for homeostatic, antibacterial, and antiviral mechanisms in the gut. PloS Pathog (2017) 13:e1006277. doi: 10.1371/journal.ppat.1006277 28472194 PMC5417715

[43] Buchon N Broderick NA Lemaitre B. Gut homeostasis in a microbial world: insights from Drosophila melanogaster. Nat Rev Microbiol (2013) 11:615–26. doi: 10.1038/nrmicro3074 23893105

[44] Capo F Wilson A Di Cara F. The intestine of drosophila melanogaster: an emerging versatile model system to study intestinal epithelial homeostasis and host-microbial interactions in humans. Microorganisms (2019). doi: 10.3390/microorganisms7090336 PMC6780840 31505811

[45] Lee J-H Lee K-A Lee W-J. Microbiota, gut physiology, and insect immunity. In: Insect immunity (2017). p. 111–38. doi: 10.1016/bs.aiip.2016.11.001pp

[46] Erkosar B Leulier F. Transient adult microbiota, gut homeostasis and longevity: novel insights from the Drosophila model. FEBS Lett (2014) 588:4250–7. doi: 10.1016/j.febslet.2014.06.041 24983497

[47] Zhu C Guan F Wang C Jin LH. The protective effects of Rhodiola crenulata extracts on Drosophila melanogaster gut immunity induced by bacteria and SDS toxicity. Phytotherapy research: PTR (2014) 28:1861–6. doi: 10.1002/ptr.5215 25146450

[48] Solomon GM Dodangoda H McCarthy-Walker T Ntim-Gyakari R Newell PD. The microbiota of Drosophila suzukii influences the larval development of Drosophila melanogaster. PeerJ (2019) 7:e8097. doi: 10.7717/peerj.8097 31763075 PMC6873876

[49] Wong. #150;Yamauchi. (2016) 2020:157.

[50] Newell PD Douglas AE. Interspecies interactions determine the impact of the gut microbiota on nutrient allocation in Drosophila melanogaster. Appl Environ Microbiol (2014) 80:788–96. doi: 10.1128/aem.02742-13 PMC3911109 24242251

[51] Yamauchi T Oi A Kosakamoto H Akuzawa-Tokita Y Murakami T Mori H. Gut bacterial species distinctively impact host purine metabolites during aging in drosophila. iScience (2020) 23:101477. doi: 10.1016/j.isci.2020.101477 32916085 PMC7520893

[52] Huang JH Douglas AE. Consumption of dietary sugar by gut bacteria determines Drosophila lipid content. Biol Lett (2015) 11:20150469. doi: 10.1098/rsbl.2015.0469 26382071 PMC4614424

[53] Dobson AJ Chaston JM Newell PD Donahue L Hermann SL Sannino DR. Host genetic determinants of microbiota-dependent nutrition revealed by genome-wide analysis of Drosophila melanogaster. Nat Commun (2015) 6:6312. doi: 10.1038/ncomms7312 25692519 PMC4333721

[54] Henry Y Tarapacki P Colinet H. Larval density affects phenotype and surrounding bacterial community without altering gut microbiota in Drosophila melanogaster. FEMS Microbiol Ecol (2020) 96. doi: 10.1093/femsec/fiaa055 32221589

[55] Dodge R Jones EW Zhu H Obadia B Martinez DJ Wang C. A symbiotic physical niche in Drosophila melanogaster regulates stable association of a multi-species gut microbiota. Nat Commun (2023) 14:1557. doi: 10.1038/s41467-023-36942-x 36944617 PMC10030875

[56] Blum JE Fischer CN Miles J Handelsman J. Frequent replenishment sustains the beneficial microbiome of Drosophila melanogaster. mBio (2013) 4:e00860–00813. doi: 10.1128/mBio.00860-13 PMC3892787 24194543

[57] Labachyan KE Kiani D Sevrioukov EA Schriner SE Jafari M. The impact of Rhodiola rosea on the gut microbial community of Drosophila melanogaster. Gut Pathog (2018) 10:12. doi: 10.1186/s13099-018-0239-8 29581730 PMC5861609

[58] Ma D Storelli G Mitchell M Leulier F. Studying host-microbiota mutualism in Drosophila: Harnessing the power of gnotobiotic flies. Biomed J (2015) 38:285–93. doi: 10.4103/2319-4170.158620 26068125

[59] Lee JH Lee KA Lee WJ. Drosophila as a model system for deciphering the ‘host physiology-nutrition-microbiome’ axis. Curr Opin Insect Sci (2020) 41:112–9. doi: 10.1016/j.cois.2020.09.005 32979529

[60] Wong AC Wang QP Morimoto J Senior AM Lihoreau M Neely GG. Gut microbiota modifies olfactory-guided microbial preferences and foraging decisions in drosophila. Curr biology: CB (2017) 27:2397–2404.e2394. doi: 10.1016/j.cub.2017.07.022 28756953

[61] Silva V Palacios-Muñoz A Okray Z Adair KL Waddell S Douglas AE. The impact of the gut microbiome on memory and sleep in Drosophila. J Exp Biol (2021) 224. doi: 10.1242/jeb.233619 PMC7875489 33376141

[62] Jia Y Jin S Hu K Geng L Han C Kang R. Gut microbiome modulates Drosophila aggression through octopamine signaling. Nat Commun (2021) 12:2698. doi: 10.1038/s41467-021-23041-y 33976215 PMC8113466

[63] Douglas AE. Drosophila and its gut microbes: a model for drug-microbiome interactions. Drug Discovery Today Dis Models (2018) 28:43–9. doi: 10.1016/j.ddmod.2019.08.004 PMC7448531 32855644

[64] Grenier T Leulier F. How commensal microbes shape the physiology of Drosophila melanogaster. Curr Opin Insect Sci (2020) 41:92–9. doi: 10.1016/j.cois.2020.08.002 32836177

[65] Lesperance DN Broderick NA. Microbiomes as modulators of Drosophila melanogaster homeostasis and disease. Curr Opin Insect Sci (2020) 39:84–90. doi: 10.1016/j.cois.2020.03.003 32339931 PMC7302976

[66] Fast D Kostiuk B Foley E Pukatzki S. Commensal pathogen competition impacts host viability. Proc Natl Acad Sci United States America (2018) 115:7099–104. doi: 10.1073/pnas.1802165115 PMC6142279 29915049

[67] . doi: 10.13040/IJPSR.0975-8232.8

[68] Su W Liu J Bai P Ma B Liu W. Pathogenic fungi-induced susceptibility is mitigated by mutual Lactobacillus plantarum in the Drosophila melanogaster model. BMC Microbiol (2019) 19:302. doi: 10.1186/s12866-019-1686-1 31864308 PMC6925846

[69] Mistry R Kounatidis I Ligoxygakis P. Interaction between familial transmission and a constitutively active immune system shapes gut microbiota in drosophila melanogaster. Genetics (2017) 206:889–904. doi: 10.1534/genetics.116.190215 28413160 PMC5499193

[70] Marra A Hanson MA Kondo S Erkosar B Lemaitre B. Drosophila antimicrobial peptides and lysozymes regulate gut microbiota composition and abundance. mBio (2021) 12:e0082421. doi: 10.1128/mBio.00824-21 34253067 PMC8406169

[71] Jang S Kikuchi Y. Impact of the insect gut microbiota on ecology, evolution, and industry. Curr Opin Insect Sci (2020) 41:33–9. doi: 10.1016/j.cois.2020.06.004 32634703

[72] Li L Xie B Dong C Wang M Liu H. Can closed artificial ecosystem have an impact on insect microbial community? A case study of yellow mealworm (Tenebrio molitor L.). Ecol Eng (2016) 86:183–9. doi: 10.1016/j.ecoleng.2015.09.015

[73] Wang Y Zhang Y. Investigation of Gut-Associated Bacteria inTenebrio molitor(Coleoptera: Tenebrionidae) Larvae Using Culture-Dependent and DGGE Methods. Ann Entomological Soc America (2015) 108:941–9. doi: 10.1093/aesa/sav079

[74] Cambon M Ogier J-C Lanois A Ferdy J-B Gaudriault S. (2018). doi: 10.1101/423178

[75] An R Liu C Wang J Jia P. Recent advances in degradation of polymer plastics by insects inhabiting microorganisms. Polymers (2023). doi: 10.3390/polym15051307 PMC10007075 36904548

[76] Brandon AM Garcia AM Khlystov NA Wu WM Criddle CS. Enhanced bioavailability and microbial biodegradation of polystyrene in an enrichment derived from the gut microbiome of tenebrio molitor (Mealworm larvae). Environ Sci Technol (2021) 55:2027–36. doi: 10.1021/acs.est.0c04952 33434009

[77] Available at: https://ssm.com/abstract=4146181.

[78] Wang X Tang T. Effects of polystyrene diet on the growth and development of tenebrio molitor. Toxics (2022) 10. doi: 10.3390/toxics10100608 PMC9610515 36287887

[79] Zhong Z Nong W Xie Y Hui JHL Chu LM. Long-term effect of plastic feeding on growth and transcriptomic response of mealworms (Tenebrio molitor L.). Chemosphere (2022) 287:132063. doi: 10.1016/j.chemosphere.2021.132063 34523442

[80] Lou Y Li Y Lu B Liu Q Yang SS Liu B. Response of the yellow mealworm (Tenebrio molitor) gut microbiome to diet shifts during polystyrene and polyethylene biodegradation. J hazardous materials (2021) 416:126222. doi: 10.1016/j.jhazmat.2021.126222 34492977

[81] Tsochatzis E Berggreen IE Tedeschi F Ntrallou K Gika H Corredig M. Gut Microbiome and Degradation Product Formation during Biodegradation of Expanded Polystyrene by Mealworm Larvae under Different Feeding Strategies. Molecules (Basel Switzerland) (2021) 26. doi: 10.3390/molecules26247568 PMC8708845 34946661

[82] Orts JM Parrado J Pascual JA Orts A Cuartero J Tejada M. Polyurethane Foam Residue Biodegradation through the Tenebrio molitor Digestive Tract: Microbial Communities and Enzymatic Activity. Polymers (2022). doi: 10.3390/polym15010204 PMC9823465 36616553

[83] Ballen-Segura MA López-Ramírez NA Peña-Pascagaza PM. Tenebrio molitor and its gut bacteria growth in polystyrene (PS) presence as the sole source carbon. Universitas Scientiarum (2020) 25:37–53. doi: 10.11144/Javeriana.SC25-1.tmai

[84] Peng BY Sun Y Xiao S Chen J Zhou X Wu WM. Influence of polymer size on polystyrene biodegradation in mealworms (Tenebrio molitor): responses of depolymerization pattern, gut microbiome, and metabolome to polymers with low to ultrahigh molecular weight. Environ Sci Technol (2022) 56:17310–20. doi: 10.1021/acs.est.2c06260 36350780

[85] Available at: https://ssm.com/abstract=440977.

[86] Przemieniecki SW Kosewska A Ciesielski S Kosewska O. Changes in the gut microbiome and enzymatic profile of Tenebrio molitor larvae biodegrading cellulose, polyethylene and polystyrene waste. Environ pollut (Barking Essex: 1987) (2020) 256:113265. doi: 10.1016/j.envpol.2019.113265 31733968

[87] Dahal S Jensen AB Lecocq A. Effect of Probiotics on Tenebrio molitor Larval Development and Resistance against the Fungal Pathogen Metarhizium brunneum. Insects (2022) 13. doi: 10.3390/insects13121114 PMC9788617 36555024

[88] Lecocq A Natsopoulou ME Berggreen IE Eilenberg J Heckmann LHL Nielsen HV. Probiotic properties of an indigenous Pediococcus pentosaceus strain on Tenebrio molitor larval growth and survival. J Insects as Food Feed (2021) 7:975–86. doi: 10.3920/jiff2020.0156

[89] Carvalho NM Teixeira F Silva S Madureira AR Pintado ME. Potential prebiotic activity of Tenebrio molitor insect flour using an optimized. Vitro gut microbiota Model Food Funct (2019) 10:3909–22. doi: 10.1039/c8fo01536h 31192321

[90] Poveda J Jiménez-Gómez A Saati-Santamaría Z Usategui-Martín R Rivas R García-Fraile P. Mealworm frass as a potential biofertilizer and abiotic stress tolerance-inductor in plants. Appl Soil Ecol (2019) 142:110–22. doi: 10.1016/j.apsoil.2019.04.016

[91] Slowik AR Herren P Bessette E Lim FS Hernández-Pelegrín L Savio C. Harmful and beneficial symbionts of Tenebrio molitor and their implications for disease management. J Insects as Food Feed (2023). doi: 10.3920/jiff2022.0171

[92] Fredensborg BL Fossdal Í Kálvalíð I Johannesen TB Stensvold CR Nielsen HV Kapel CMO. Parasites modulate the gut-microbiome in insects: A proof-of-concept study. PloS One (2020) 15:e0227561. doi: 10.1371/journal.pone.0227561 31935259 PMC6959588

[93] Vigneron A Jehan C Rigaud T Moret Y. Immune defenses of a beneficial pest: the mealworm beetle, tenebrio molitor. Front Physiol (2019) 10:138. doi: 10.3389/fphys.2019.00138 30914960 PMC6422893

[94] Ali Mohammadie Kojour M Han YS Jo YH. An overview of insect innate immunity. Entomological Res (2020) 50:282–91. doi: 10.1111/1748-5967.12437

[95] Lu Y Su F Li Q Zhang J Li Y Tang T. Pattern recognition receptors in Drosophila immune responses. Dev Comp Immunol (2020) 102:103468. 31430488 10.1016/j.dci.2019.103468

[96] El Chamy L Matt N Ntwasa M Reichhart JM. The multilayered innate immune defense of the gut. Biomed J (2015) 38:276–84. doi: 10.4103/2319-4170.158621 26068126

[97] Myllymaki H Valanne S Ramet M. The Drosophila imd signaling pathway. J Immunol (2014) 192:3455–62. doi: 10.4049/jimmunol.1303309 24706930

[98] Zhai Z Boquete JP Lemaitre B. Cell-Specific Imd-NF-κB Responses Enable Simultaneous Antibacterial Immunity and Intestinal Epithelial Cell Shedding upon Bacterial Infection. Immunity (2018) 48:897–910.e897. doi: 10.1016/j.immuni.2018.04.010 29752064

[99] Kamareddine L Robins WP Berkey CD Mekalanos JJ Watnick PI. The Drosophila Immune Deficiency Pathway Modulates Enteroendocrine Function and Host Metabolism. Cell Metab (2018) 28:449–462.e445. doi: 10.1016/j.cmet.2018.05.026 29937377 PMC6125180

[100] Bosco-Drayon V Poidevin M Boneca IG Narbonne-Reveau K Royet J Charroux B. Peptidoglycan sensing by the receptor PGRP-LE in the Drosophila gut induces immune responses to infectious bacteria and tolerance to microbiota. Cell Host Microbe (2012) 12:153–65. doi: 10.1016/j.chom.2012.06.002 22901536

[101] Wen Y He Z Xu T Jiao Y Liu X Wang YF. Ingestion of killed bacteria activates antimicrobial peptide genes in Drosophila melanogaster and protects flies from septic infection. Dev Comp Immunol (2019) 95:10–8. doi: 10.1016/j.dci.2019.02.001 30731096

[102] Ferrandon D. The complementary facets of epithelial host defenses in the genetic model organism Drosophila melanogaster: from resistance to resilience. Curr Opin Immunol (2013) 25:59–70. doi: 10.1016/j.coi.2012.11.008 23228366

[103] Limmer S Haller S Drenkard E Lee J Yu S Kocks C. Pseudomonas aeruginosa RhlR is required to neutralize the cellular immune response in a Drosophila melanogaster oral infection model. Proc Natl Acad Sci United States America (2011) 108:17378–83. doi: 10.1073/pnas.1114907108 PMC3198323 21987808

[104] Hori A Kurata S Kuraishi T. Unexpected role of the IMD pathway in Drosophila gut defense against Staphylococcus aureus. Biochem Biophys Res Commun (2018) 495:395–400. doi: 10.1016/j.bbrc.2017.11.004 29108998

[105] Watnick PI Jugder BE. Microbial Control of Intestinal Homeostasis *via* Enteroendocrine Cell Innate Immune Signaling. Trends Microbiol (2020) 28:141–9. doi: 10.1016/j.tim.2019.09.005 PMC6980660 31699645

[106] Jugder BE Kamareddine L Watnick PI. Microbiota-derived acetate activates intestinal innate immunity *via* the Tip60 histone acetyltransferase complex. Immunity (2021) 54:1683–1697.e1683. doi: 10.1016/j.immuni.2021.05.017 34107298 PMC8363570

[107] Kosakamoto H Yamauchi T Akuzawa-Tokita Y Nishimura K Soga T Murakami T. Local Necrotic Cells Trigger Systemic Immune Activation *via* Gut Microbiome Dysbiosis in Drosophila. Cell Rep (2020) 32:107938. doi: 10.1016/j.celrep.2020.107938 32698005

[108] Wang Z Hang S Purdy AE Watnick PI. Mutations in the IMD pathway and mustard counter Vibrio cholerae suppression of intestinal stem cell division in Drosophila. mBio (2013) 4:e00337–00313. doi: 10.1128/mBio.00337-13 PMC3684835 23781070

[109] Jang HA Kojour MAM Patnaik BB Han YS Jo YH. Current status of immune deficiency pathway in Tenebrio molitor innate immunity. Front Immunol (2022) 13:906192. 35860244 10.3389/fimmu.2022.906192 PMC9292131

[110] Kleino A Silverman N. The Drosophila IMD pathway in the activation of the humoral immune response. Dev Comp Immunol (2014) 42:25–35. 23721820 10.1016/j.dci.2013.05.014 PMC3808521

[111] Ali Mohammadie Kojour M Edosa TT Jang HA Keshavarz M Jo YH Han YS. Critical roles of spätzle5 in antimicrobial peptide production against Escherichia coli in Tenebrio molitor malpighian tubules. Front Immunol (2021) 12:760475. 34975850 10.3389/fimmu.2021.760475 PMC8717915

[112] Chowdhury M Li C-F He Z Lu Y Liu X-S Wang Y-F. Toll family members bind multiple Spätzle proteins and activate antimicrobial peptide gene expression in Drosophila. J Biol Chem (2019) 294:10172–81. 10.1074/jbc.RA118.006804 PMC6664172 31088910

[113] Edosa TT Jo YH Keshavarz M Bae YM Kim DH Lee YS. Tm Spz4 plays an important role in regulating the production of antimicrobial peptides in response to Escherichia coli and Candida albicans infections. Int J Mol Sci (2020) 21:1878. 32182940 10.3390/ijms21051878 PMC7084639

[114] Edosa TT Jo YH Keshavarz M Bae YM Kim DH Lee YS. Tm Spz6 is essential for regulating the immune response to Escherichia coli and Staphylococcus aureus infection in Tenebrio molitor. Insects (2020) 11:105. 32033290 10.3390/insects11020105 PMC7074004

[115] Edosa TT Jo YH Keshavarz M Kim IS Han YS. Biosurfactants Induce Antimicrobial Peptide Production through the Activation of Tm Spatzles in Tenebrio molitor. Int J Mol Sci (2020) 21:6090. 32847078 10.3390/ijms21176090 PMC7504391

[116] Jang HA Patnaik BB Ali Mohammadie Kojour M Kim BB Bae YM Park KB. Tm Spz-like plays a fundamental role in response to E. coli but not S. aureus or C. albican infection in Tenebrio molitor *via* regulation of antimicrobial peptide production. Int J Mol Sci (2021) 22:10888. 34639230 10.3390/ijms221910888 PMC8509142

[117] Kambris Z Brun S Jang I-H Nam H-J Romeo Y Takahashi K. Drosophila immunity: a large-scale *in vivo* RNAi screen identifies five serine proteases required for Toll activation. Curr Biol (2006) 16:808–13. 10.1016/j.cub.2006.03.020 16631589

[118] Ali Mohammadie Kojour M Jang HA Lee YS Jo YH Han YS. Immunological Roles of TmToll-2 in Response to Escherichia coli Systemic Infection in Tenebrio molitor. Int J Mol Sci (2022) 23:14490. 36430968 10.3390/ijms232214490 PMC9699188

[119] Ali Mohammadie Kojour M Jang HA Lee YS Jo YH Han YS. Innate Immune Response of TmToll-3 Following Systemic Microbial Infection in Tenebrio molitor. Int J Mol Sci (2023) 24:6751. 37047723 10.3390/ijms24076751 PMC10095136

[120] Tanji T Hu X Weber AN Ip YT. Toll and IMD pathways synergistically activate an innate immune response in Drosophila melanogaster. Mol Cell Biol (2007) 27:4578–88. 10.1128/MCB.01814-06 PMC1900069 17438142

[121] Yagi Y Nishida Y Ip YT. Functional analysis of Toll-related genes in Drosophila. Development Growth differentiation (2010) 52:771–83. 10.1111/j.1440-169X.2010.01213.x PMC4017902 21158756

[122] Jo YH Kim YJ Park KB Seong JH Kim SG Park S. TmCactin plays an important role in Gram-negative and-positive bacterial infection by regulating expression of 7 AMP genes in Tenebrio molitor. Sci Rep (2017) 7:46459. 28418029 10.1038/srep46459 PMC5394457

[123] Patnaik BB Patnaik HH Seo GW Jo YH Lee YS Lee BL. Gene structure, cDNA characterization and RNAi-based functional analysis of a myeloid differentiation factor 88 homolog in Tenebrio molitor larvae exposed to Staphylococcus aureus infection. Dev Comp Immunol (2014) 46:208–21. 10.1016/j.dci.2014.04.009 24755285

[124] Keshavarz M Jo YH Park KB Ko HJ Edosa TT Lee YS. Tm DorX2 positively regulates antimicrobial peptides in Tenebrio molitor gut, fat body, and hemocytes in response to bacterial and fungal infection. Sci Rep (2019) 9:16878. 31728023 10.1038/s41598-019-53497-4 PMC6856108

[125] Combe BE Defaye A Bozonnet N Puthier D Royet J Leulier F. Drosophila microbiota modulates host metabolic gene expression *via* IMD/NF-κB signaling. PloS One (2014) 9:e94729. 24733183 10.1371/journal.pone.0094729 PMC3986221

[126] Tapadia MG Verma P. Immune response and anti-microbial peptides expression in Malpighian tubules of Drosophila melanogaster is under developmental regulation. PloS One (2012) 7:e40714. 22808242 10.1371/journal.pone.0040714 PMC3395640

[127] Ko HJ Am Jang H Park KB Kim CE Patnaik BB Lee YS. IKKβ regulates antimicrobial innate immune responses in the yellow mealworm, Tenebrio molitor. Dev Comp Immunol (2023) 104761. 10.1016/j.dci.2023.104761 37331676

[128] Schmidt RL Trejo TR Plummer TB Platt JL Tang AH. Infection-induced proteolysis of PGRP-LC controls the IMD activation and melanization cascades in Drosophila. FASEB J (2008) 22:918–29. 10.1096/fj.06-7907com 18308747

[129] Wen Y He Z Xu T Jiao Y Liu X Wang Y-F. Ingestion of killed bacteria activates antimicrobial peptide genes in Drosophila melanogaster and protects flies from septic infection. Dev Comp Immunol (2019) 95:10–8. 10.1016/j.dci.2019.02.001 30731096

[130] Jang HA Park KB Kim BB Ali Mohammadie Kojour M Bae YM Baliarsingh S. In silico identification and expression analyses of Defensin genes in the mealworm beetle Tenebrio molitor. Entomological Res (2020) 50:575–85.

[131] Keshavarz M Jo YH Edosa TT Bae YM Han YS. Tm PGRP-SA regulates antimicrobial response to bacteria and fungi in the fat body and gut of Tenebrio molitor. Int J Mol Sci (2020) 21:2113. 32204438 10.3390/ijms21062113 PMC7139795

[132] Kambris Z Hoffmann JA Imler J-L Capovilla M. Tissue and stage-specific expression of the Tolls in Drosophila embryos. Gene Expression Patterns (2002) 2:311–7. 10.1016/s1567-133x(02)00020-0 12617819

[133] Park S Jo YH Park KB Ko HJ Kim CE Bae YM. TmToll-7 plays a crucial role in innate immune responses against Gram-negative bacteria by regulating 5 AMP genes in Tenebrio molitor. Front Immunol (2019) 10:310. 30930888 10.3389/fimmu.2019.00310 PMC6424196

[134] Yang YT Lee MR Lee SJ Kim S Nai YS Kim JS. Tenebrio molitor Gram-negative-binding protein 3 (TmGNBP3) is essential for inducing downstream antifungal Tenecin 1 gene expression against infection with Beauveria bassiana JEF-007. Insect Sci (2018) 25:969–77. 10.1111/1744-7917.12482 28544681

[135] Ali Mohammadie Kojour M Edosa TT Jang HA Keshavarz M Jo YH Han YS. Critical Roles of Spätzle5 in Antimicrobial Peptide Production Against Escherichia coli in Tenebrio molitor Malpighian Tubules. Front Immunol (2021) 12:760475. doi: 10.3389/fimmu.2021.760475 34975850 PMC8717915

[136] Ko HJ Jo YH Patnaik BB Park KB Kim CE Keshavarz M. IKKγ/NEMO Is Required to Confer Antimicrobial Innate Immune Responses in the Yellow Mealworm, Tenebrio Molitor. Int J Mol Sci 2020 21. doi: 10.3390/ijms21186734 PMC7555931 32937897

[137] Jang HA Kojour MAM Patnaik BB Han YS Jo YH. Current Status of Immune Deficiency Pathway in Tenebrio molitor Innate Immunity. Front Immunol (2022) 13:906192. doi: 10.3389/fimmu.2022.906192 35860244 PMC9292131

[138] Jo YH Patnaik BB Hwang J Park KB Ko HJ Kim CE. Regulation of the expression of nine antimicrobial peptide genes by TmIMD confers resistance against Gram-negative bacteria. Sci Rep (2019) 9:10138. doi: 10.1038/s41598-019-46222-8 31300668 PMC6626034

[139] Keshavarz M Jo YH Edosa TT Bae YM Han YS. TmPGRP-SA regulates Antimicrobial Response to Bacteria and Fungi in the Fat Body and Gut of Tenebrio molitor. Int J Mol Sci (2020) 21. doi: 10.3390/ijms21062113 PMC7139795 32204438

[140] Keshavarz M Jo YH Edosa TT Han YS. Tenebrio molitor PGRP-LE Plays a Critical Role in Gut Antimicrobial Peptide Production in Response to Escherichia coli. Front Physiol (2020) 11:320. doi: 10.3389/fphys.2020.00320 32372972 PMC7179671

[141] Keshavarz M Jo YH Patnaik BB Park KB Ko HJ Kim CE. TmRelish is required for regulating the antimicrobial responses to Escherichia coli and Staphylococcus aureus in Tenebrio molitor. Sci Rep (2020) 10:4258. doi: 10.1038/s41598-020-61157-1 32144366 PMC7060202

[142] Lemaitre B Miguel-Aliaga I. The digestive tract of Drosophila melanogaster. Annu Rev Genet (2013) 47:377–404. doi: 10.1146/annurev-genet-111212-133343 24016187

[143] Myllymaki H Ramet M. JAK/STAT pathway in Drosophila immunity. Scand J Immunol (2014) 79:377–85. doi: 10.1111/sji.12170 24673174

[144] Kietz C Meinander A. Drosophila caspases as guardians of host-microbe interactions. Cell Death Differentiation (2023) 30:227–36. 10.1038/s41418-022-01038-4 PMC9950452 35810247

[145] Liu X Nagy P Bonfini A Houtz P Bing XL Yang X. Microbes affect gut epithelial cell composition through immune-dependent regulation of intestinal stem cell differentiation. Cell Rep (2022) 38:110572. doi: 10.1016/j.celrep.2022.110572 35354023 PMC9078081

[146] Buchon N Broderick NA Lemaitre B. Gut homeostasis in a microbial world: insights from Drosophila melanogaster. Nat Rev Microbiol (2013) 11:615–26. 10.1038/nrmicro3074 23893105

[147] Charroux B Royet J. Drosophila immune response: From systemic antimicrobial peptide production in fat body cells to local defense in the intestinal tract. Fly (Austin) (2010) 4:40–7. doi: 10.4161/fly.4.1.10810 20383054

[148] You H Lee WJ Lee WJ. Homeostasis between gut-associated microorganisms and the immune system in Drosophila. Curr Opin Immunol (2014) 30:48–53. doi: 10.1016/j.coi.2014.06.006 24997434

[149] Ha EM Lee KA Park SH Kim SH Nam HJ Lee HY. Regulation of DUOX by the Galphaq-phospholipase Cbeta-Ca2+ pathway in Drosophila gut immunity. Dev Cell (2009) 16:386–97. doi: 10.1016/j.devcel.2008.12.015 19289084

[150] Lee KA Kim B Bhin J Kim DH You H Kim EK. Bacterial uracil modulates Drosophila DUOX-dependent gut immunity *via* Hedgehog-induced signaling endosomes. Cell Host Microbe (2015) 17:191–204. doi: 10.1016/j.chom.2014.12.012 25639794

[151] Lee KA Cho KC Kim B Jang IH Nam K Kwon YE. Inflammation-Modulated Metabolic Reprogramming Is Required for DUOX-Dependent Gut Immunity in Drosophila. Cell Host Microbe (2018) 23:338–352.e335. doi: 10.1016/j.chom.2018.01.011 29503179

[152] Xiao X Yang L Pang X Zhang R Zhu Y Wang P. Mesh-Duox pathway regulates homeostasis in the insect gut. Nat Microbiol (2017) 2:17020. doi: 10.1038/nmicrobiol.2017.20 28248301 PMC5332881

[153] Lee KA Kim SH Kim EK Ha EM You H Kim B. Bacterial-derived uracil as a modulator of mucosal immunity and gut-microbe homeostasis in Drosophila. Cell (2013) 153:797–811. doi: 10.1016/j.cell.2013.04.009 23663779

[154] Tafesh-Edwards G Eleftherianos I. JNK signaling in Drosophila immunity and homeostasis. Immunol Lett (2020) 226:7–11. 32598968 10.1016/j.imlet.2020.06.017

[155] Tafesh-Edwards G Eleftherianos I. JNK signaling in Drosophila immunity and homeostasis. Immunol Lett (2020) 226:7–11. doi: 10.1016/j.imlet.2020.06.017 32598968

[156] Zhang X Jin Q Jin LH. High sugar diet disrupts gut homeostasis though JNK and STAT pathways in Drosophila. Biochem Biophys Res Commun (2017) 487:910–6. doi: 10.1016/j.bbrc.2017.04.156 28476621

[157] Ahn HM Lee KS Lee DS Yu K. JNK/FOXO mediated PeroxiredoxinV expression regulates redox homeostasis during Drosophila melanogaster gut infection. Dev Comp Immunol (2012) 38:466–73. doi: 10.1016/j.dci.2012.07.002 22858408

[158] Sansone CL Cohen J Yasunaga A Xu J Osborn G Subramanian H. Microbiota-Dependent Priming of Antiviral Intestinal Immunity in Drosophila. Cell Host Microbe (2015) 18:571–81. doi: 10.1016/j.chom.2015.10.010 PMC4648705 26567510

[159] Chen J Xie C Tian L Hong L Wu X Han J. Participation of the p38 pathway in Drosophila host defense against pathogenic bacteria and fungi. Proc Natl Acad Sci United States America (2010) 107:20774–9. doi: 10.1073/pnas.1009223107 PMC2996427 21076039

[160] Ryu K Park J Kurokawa K Matsushita M Lee B. The molecular activation and regulation mechanisms of proteolytic Toll signaling cascade in insect innate immunity. Invertebrate Survival J (2010) 7:181–91.

[161] Kietz C Meinander A. Drosophila caspases as guardians of host-microbe interactions. Cell Death differentiation (2023) 30:227–36. doi: 10.1038/s41418-022-01038-4 PMC9950452 35810247

[162] Jang HA Patnaik BB Ali Mohammadie Kojour M Kim BB Bae YM Park KB. TmSpz-like Plays a Fundamental Role in Response to E. coli but Not S. aureus or C. albican Infection in Tenebrio molitor *via* Regulation of Antimicrobial Peptide Production. Int J Mol Sci (2021) 22. doi: 10.3390/ijms221910888 PMC8509142 34639230

[163] Jang HA Park KB Kim BB Ali Mohammadie Kojour M Bae YM Baliarsingh S. Bacterial but not fungal challenge up-regulates the transcription of Coleoptericin genes in Tenebrio molitor. Entomological Res (2020) 50:440–9. doi: 10.1111/1748-5967.12465

[164] Ali Mohammadie Kojour M Jang HA Lee YS Jo YH Han YS. Immunological Roles of TmToll-2 in Response to Escherichia coli Systemic Infection in Tenebrio molitor. Int J Mol Sci (2022) 23. doi: 10.3390/ijms232214490 PMC9699188 36430968

[165] Ali Mohammadie Kojour M Jang HA Lee YS Jo YH Han YS. Innate Immune Response of TmToll-3 Following Systemic Microbial Infection in Tenebrio molitor. Int J Mol Sci 2023 24. doi: 10.3390/ijms24076751 PMC10095136 37047723

[166] Nászai M Carroll LR Cordero JB. Intestinal stem cell proliferation and epithelial homeostasis in the adult Drosophila midgut. Insect Biochem Mol Biol (2015) 67:9–14. doi: 10.1016/j.ibmb.2015.05.016 26024801

[167] Lee J-H Lee K-A Lee W-J. Microbiota, gut physiology, and insect immunity. Adv Insect Physiol (2017) 52:111–38.

[168] Buchon N Broderick NA Chakrabarti S Lemaitre B. Invasive and indigenous microbiota impact intestinal stem cell activity through multiple pathways in Drosophila. Genes Dev (2009) 23:2333–44. doi: 10.1101/gad.1827009 PMC2758745 19797770

[169] Chakrabarti S Liehl P Buchon N Lemaitre B. Infection-induced host translational blockage inhibits immune responses and epithelial renewal in the Drosophila gut. Cell Host Microbe (2012) 12:60–70. doi: 10.1016/j.chom.2012.06.001 22817988

[170] Jiang H Patel PH Kohlmaier A Grenley MO McEwen DG Edgar BA. Cytokine/Jak/Stat signaling mediates regeneration and homeostasis in the Drosophila midgut. Cell (2009) 137:1343–55. doi: 10.1016/j.cell.2009.05.014 PMC2753793 19563763

[171] Buchon N Broderick NA Kuraishi T Lemaitre B. Drosophila EGFR pathway coordinates stem cell proliferation and gut remodeling following infection. BMC Biol (2010) 8:152. doi: 10.1186/1741-7007-8-152 21176204 PMC3022776

[172] Xiang J Bandura J Zhang P Jin Y Reuter H Edgar BA. EGFR-dependent TOR-independent endocycles support Drosophila gut epithelial regeneration. Nat Commun (2017) 8:15125. doi: 10.1038/ncomms15125 28485389 PMC5436070

[173] Shaw RL Kohlmaier A Polesello C Veelken C Edgar BA Tapon N. The Hippo pathway regulates intestinal stem cell proliferation during Drosophila adult midgut regeneration. Development (2010) 137:4147–58. doi: 10.1242/dev.052506 PMC2990206 21068063

[174] Fan X Gaur U Yang M. Intestinal Homeostasis and Longevity: Drosophila Gut Feeling. Adv Exp Med Biol (2018) 1086:157–68. doi: 10.1007/978-981-13-1117-8_10 30232758

